# Supplementary material for: Synthesis and Biological Evaluation of Diversified Hamigeran B Analogs as Neuroinflammatory Inhibitors and Neurite Outgrowth Stimulators
Source: Mar Drugs. 2020 Jun 11;18(6):306. doi: 10.3390/md18060306 (PMC7345552; doi:10.3390/md18060306)

Supplementary Materials

Synthesis and Biological Evaluation of Diversified Hamigeran B Analogs as Neuroinflammatory Inhibitors and Neurite Outgrowth Stimulators

Ruo-Xin Li [1], Rui Han [1], Guo-Jie Wu [2], Fu-She Han [2,*] and Jin-Ming Gao [1,*]

[1] Shaanxi Province Key Laboratory of Natural Products & Chemical Biology, College of Chemistry & Pharmacy, Northwest A&F University, Yangling 712100, Shaanxi, People's Republic of China; jinminggao@nwsuaf.edu.cn (J.M.G); rxli2014@hotmail.com (R.X.L); hanrui0820@nwsuaf.edu.cn (R.H)

[2] Jilin Province Key Laboratory of Green Chemistry and Process, Changchun Institute of Applied Chemistry, Chinese Academy of Sciences, 5625 Renmin Street, Changchun 130022, Jilin, People's Republic of China; fshan@ciac.ac.cn (F.S.H); gjwu@ciac.ac.cn (G.J.W);

* Correspondence: jinminggao@nwsuaf.edu.cn; fshan@ciac.ac.cn; Tel/ Fax: + 86-29-87092335 (J.M.G).

Table of Contents

1. The IC_{50} concentration curves ……………………………………………………..…..S1

2. Date of biological activity………………………………………………………………S2

3. NMR Spectra……………………………………………………………………….…....S3

1. The IC_{50} concentration curves

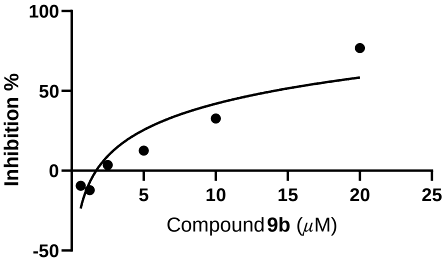

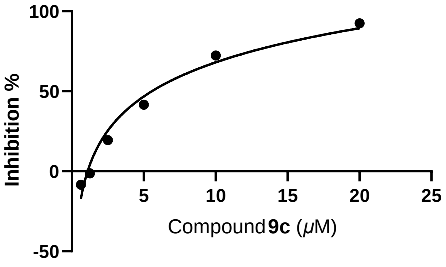

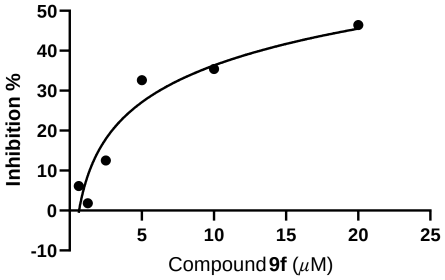

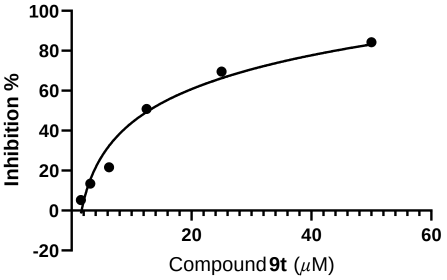

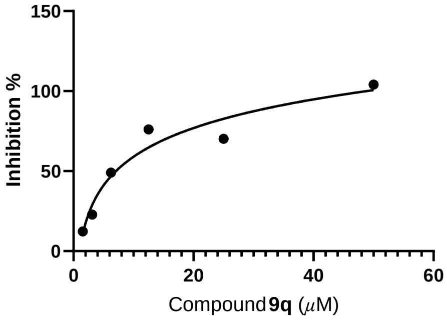

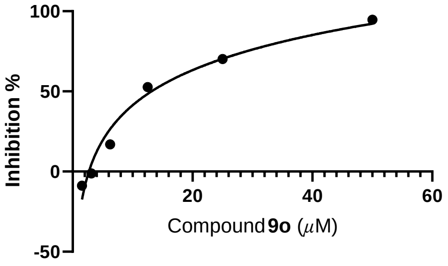

2. Date of biological activity

Table 1. Percentage of neurite-bearing PC12 cells and inhibitory effects in BV-2 cells of compounds

Compounds	Neurite-bearing cells (%)	Inflammatory inhibition (%)
NGF	9.35	--
9a	15.94	26.16
9b	11.85	41.86
9c	8.55	45.35
9d	5.56	17.44
9e	6.30	-38.37
9f	12.64	43.60
9g	11.22	10.47
9h	14.99	-45.45
9i	8.53	31.40
9j	10.60	10.47
9k	7.75	-27.91
9l	7.35	-19.19
9m	10.27	10.47
9n	10.12	38.37
9o	24.23	73.38
9p	12.83	24.42
9q	16.68	56.83
9r	11.22	-29.93
9s	9.51	-0.69
9t	12.95	61.38
9u	8.24	-70.90
9v	11.45	22.62
9w	12.09	29.38

3. NMR Spectra

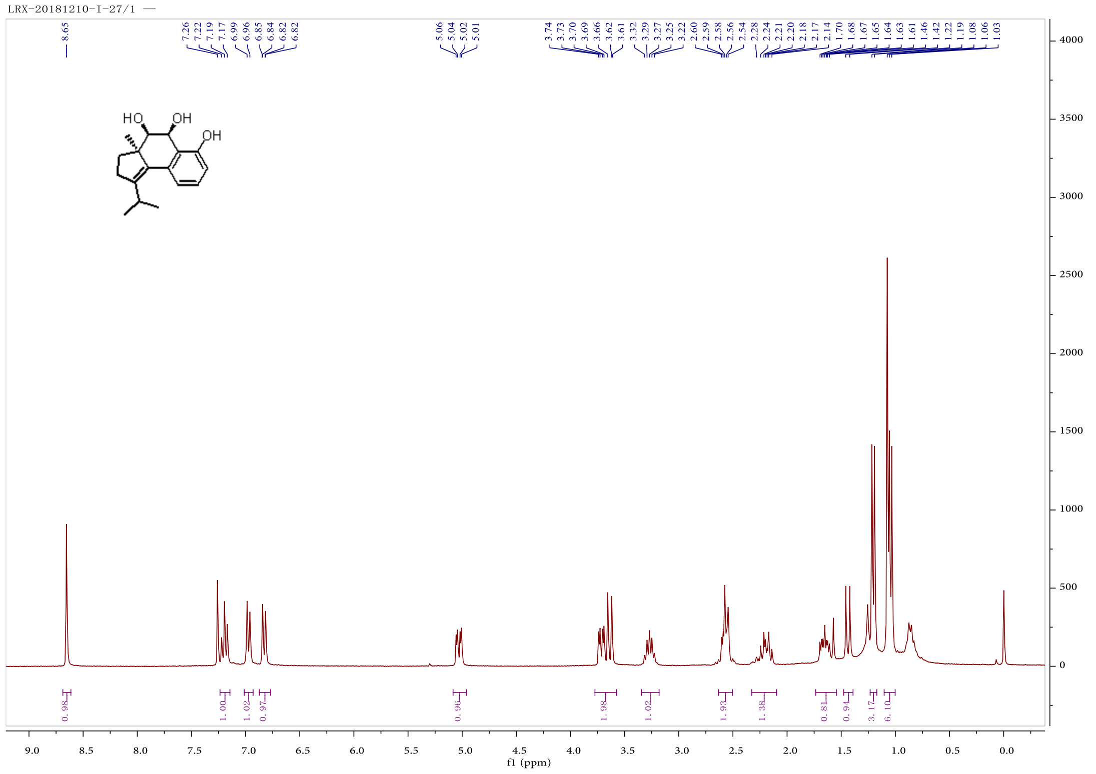

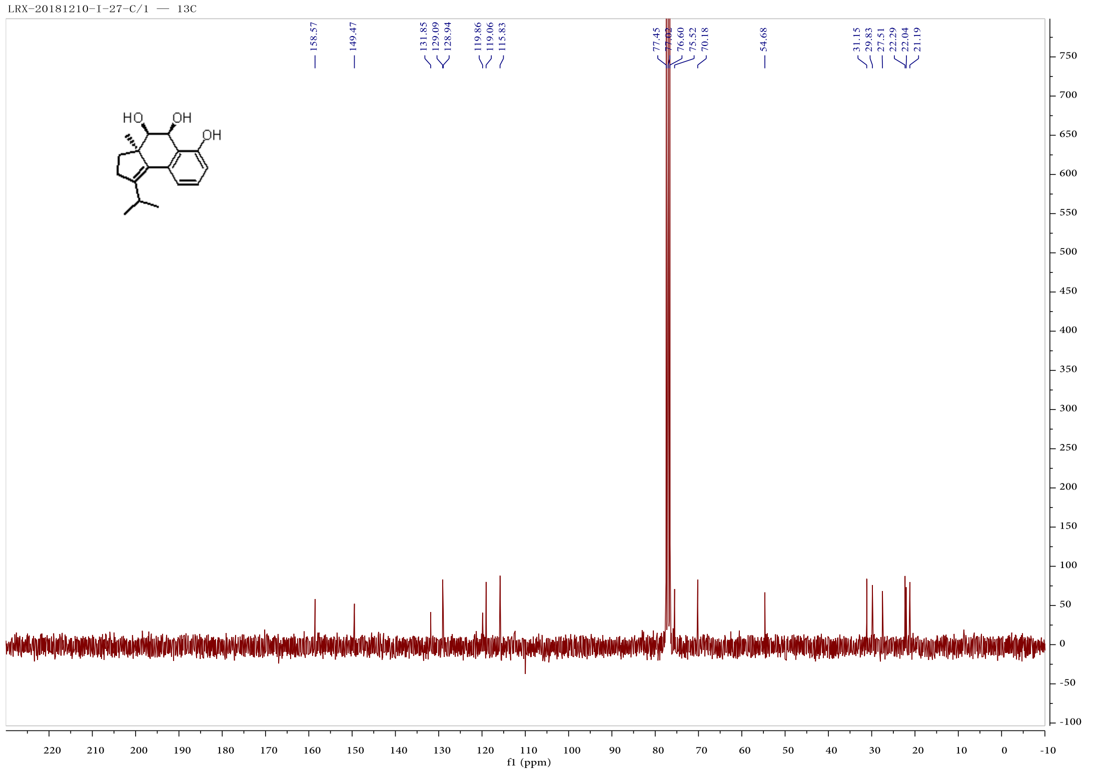

LRX-20181214-I-30/1 —

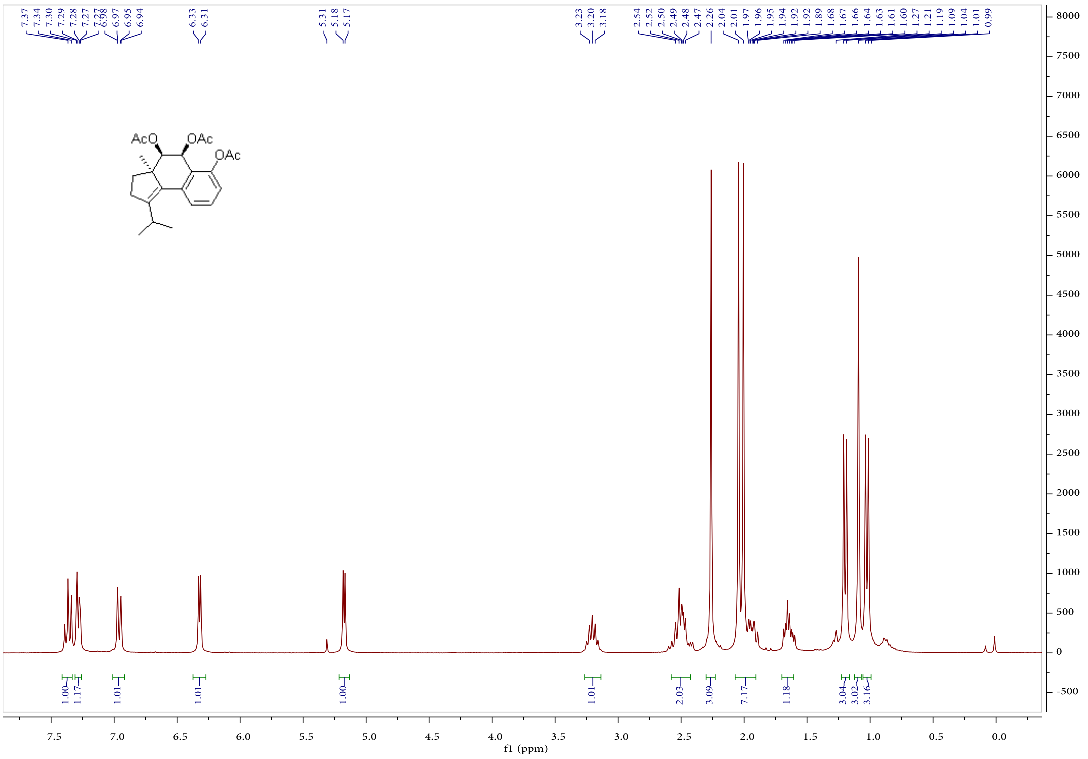

LRX-20181214-I-30-C/1 — 13C

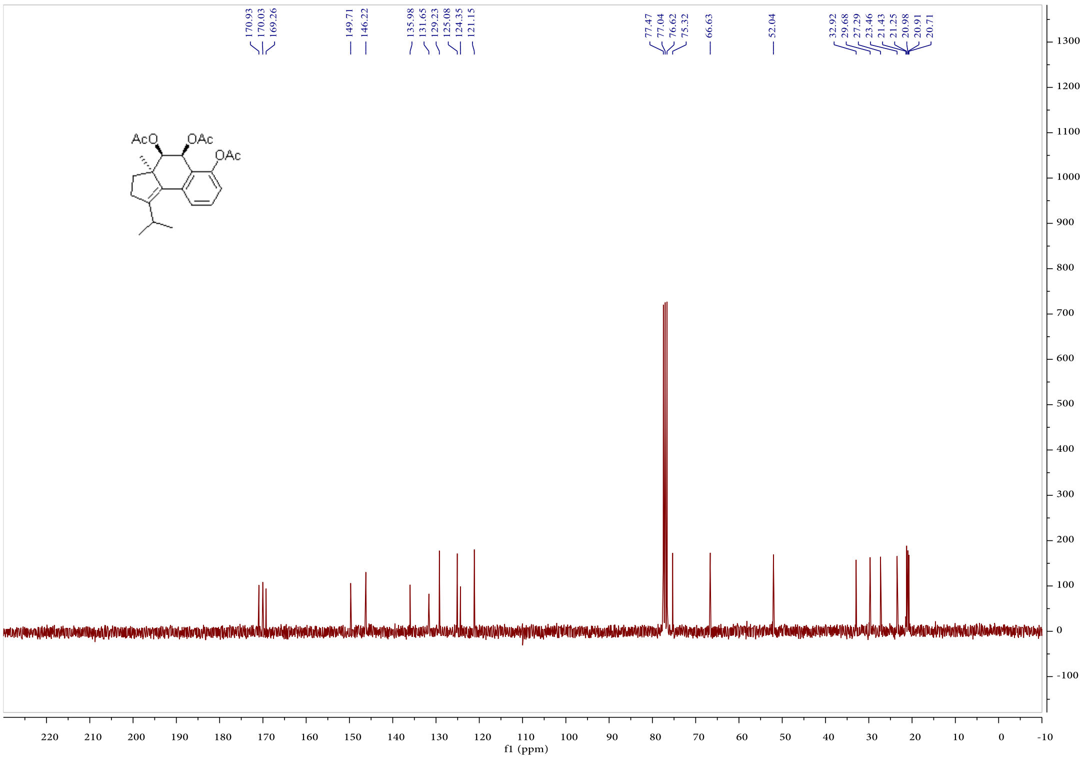

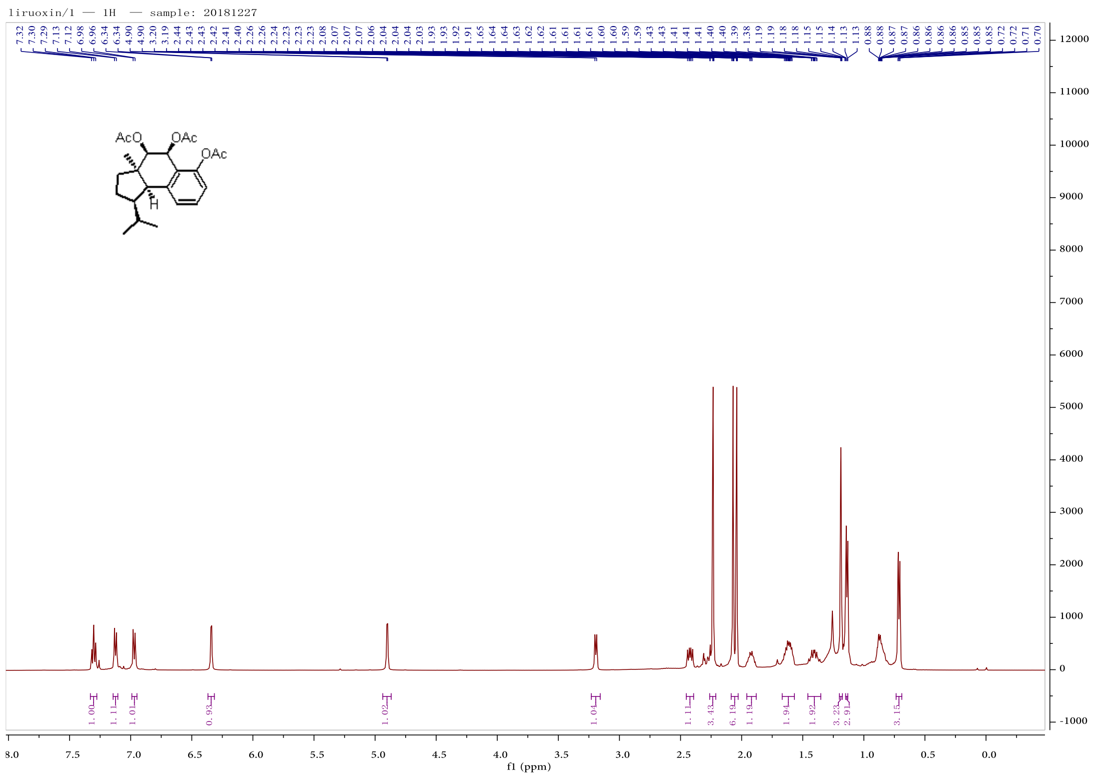
liruoxin/1 — 1H — sample: 20181227
AcO
OAc
OAc
H
f1 (ppm)

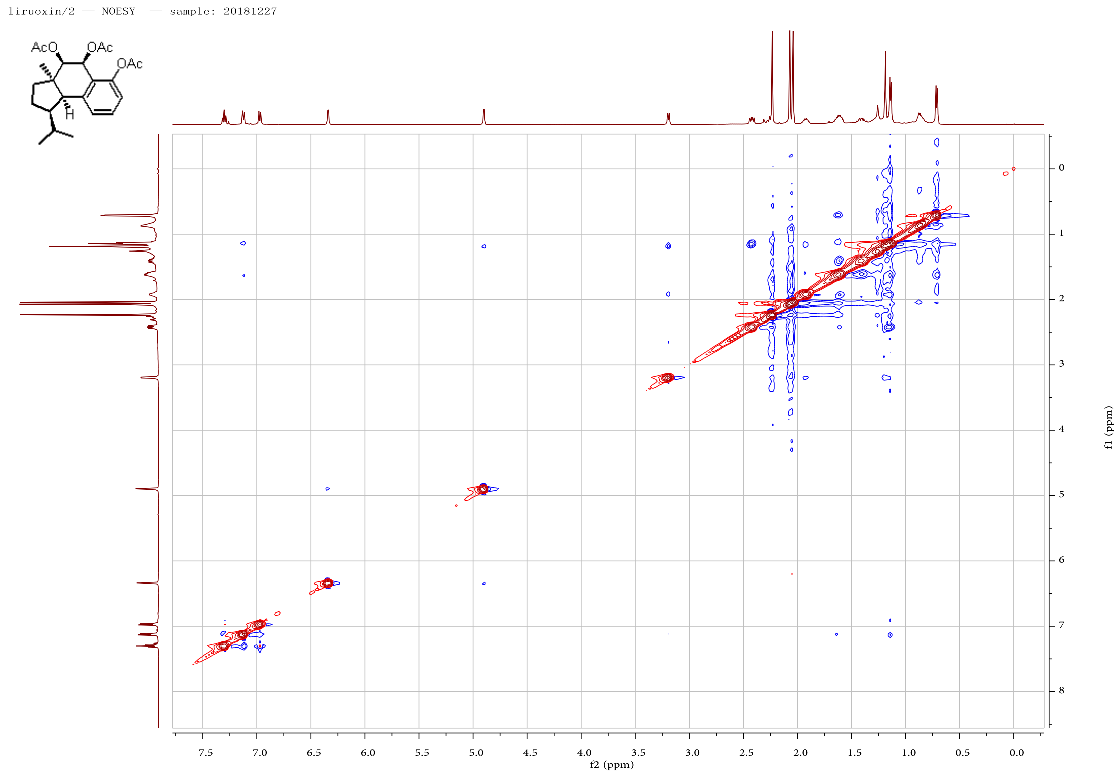
liruoxin/2 — NOESY — sample: 20181227
AcO
OAc
OAc
H
f2 (ppm)
f1 (ppm)

LRX-20181226-I-32-C/1 — 13C

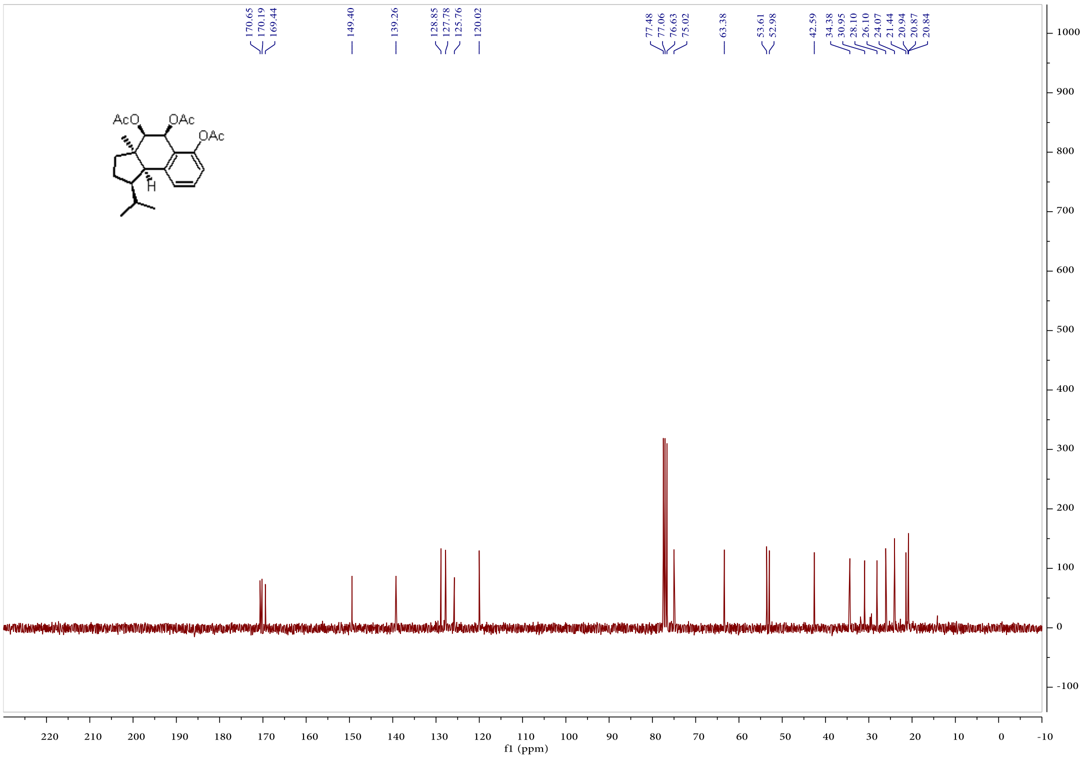

LRX-20190425-I-33/1 —

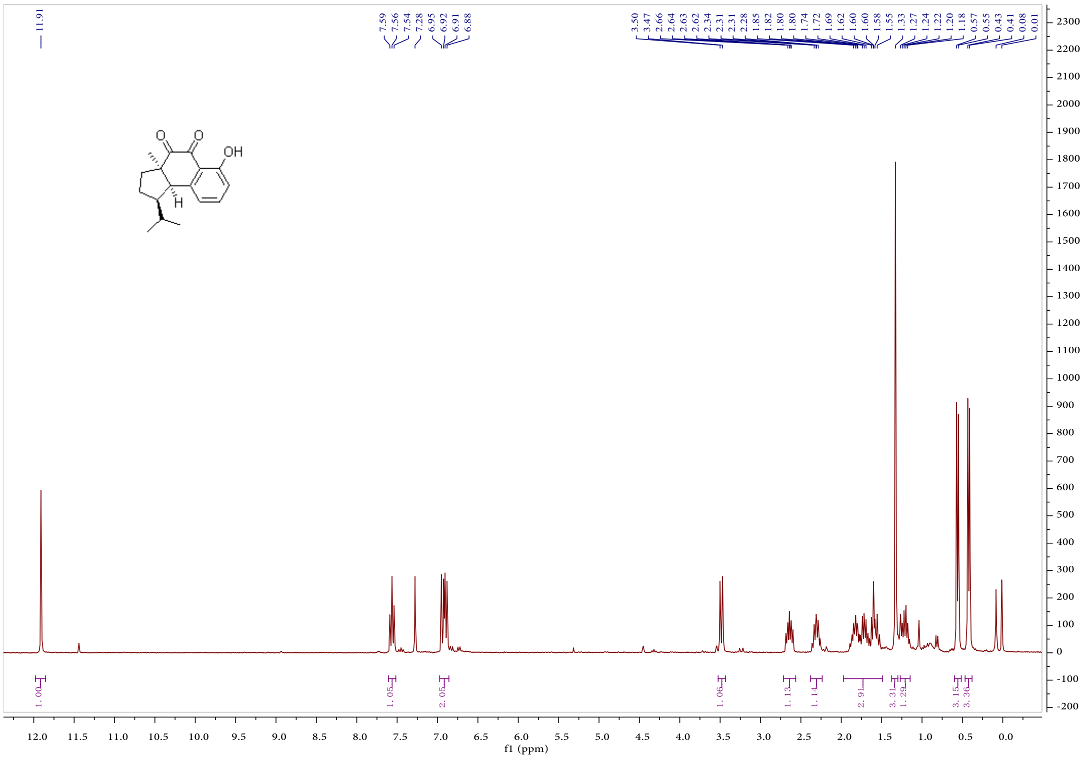

LRX-20190425-I-33-c/1 — 13C

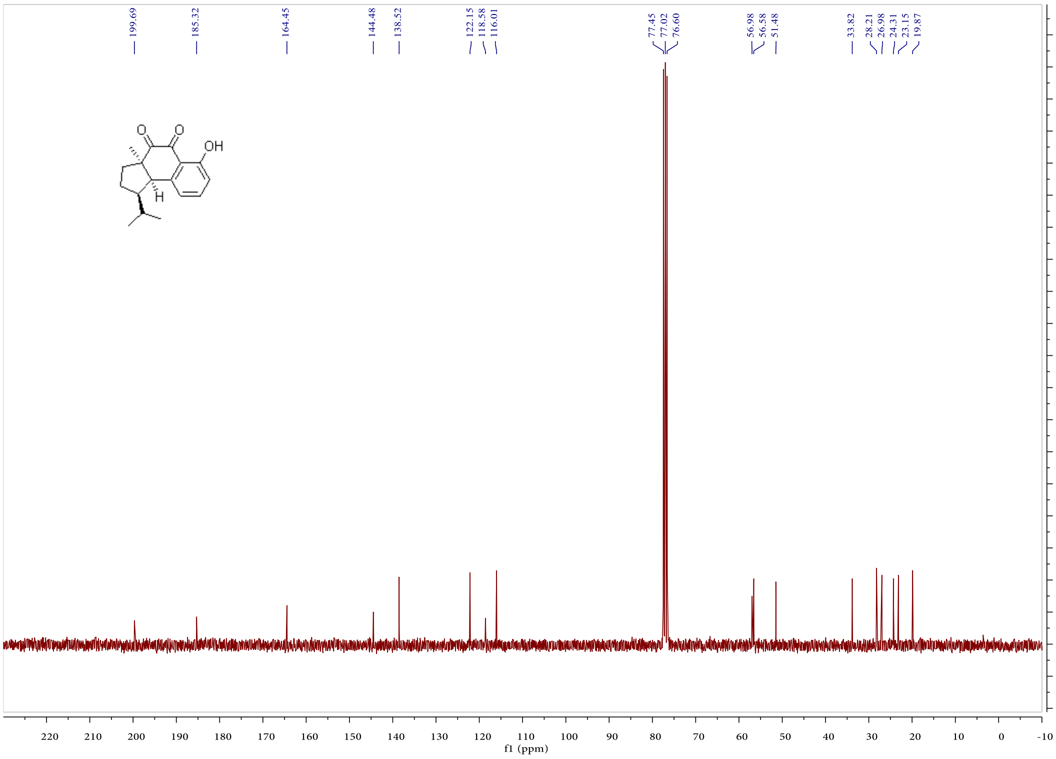

LRX-20190429-I-48/1 —

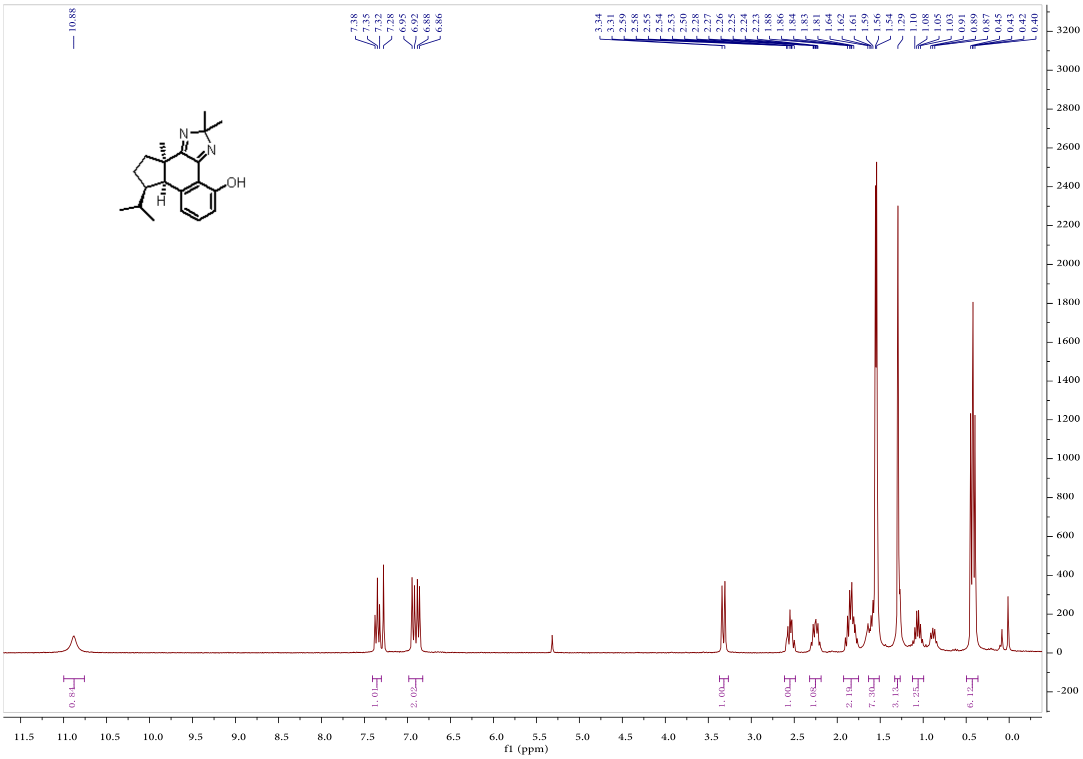

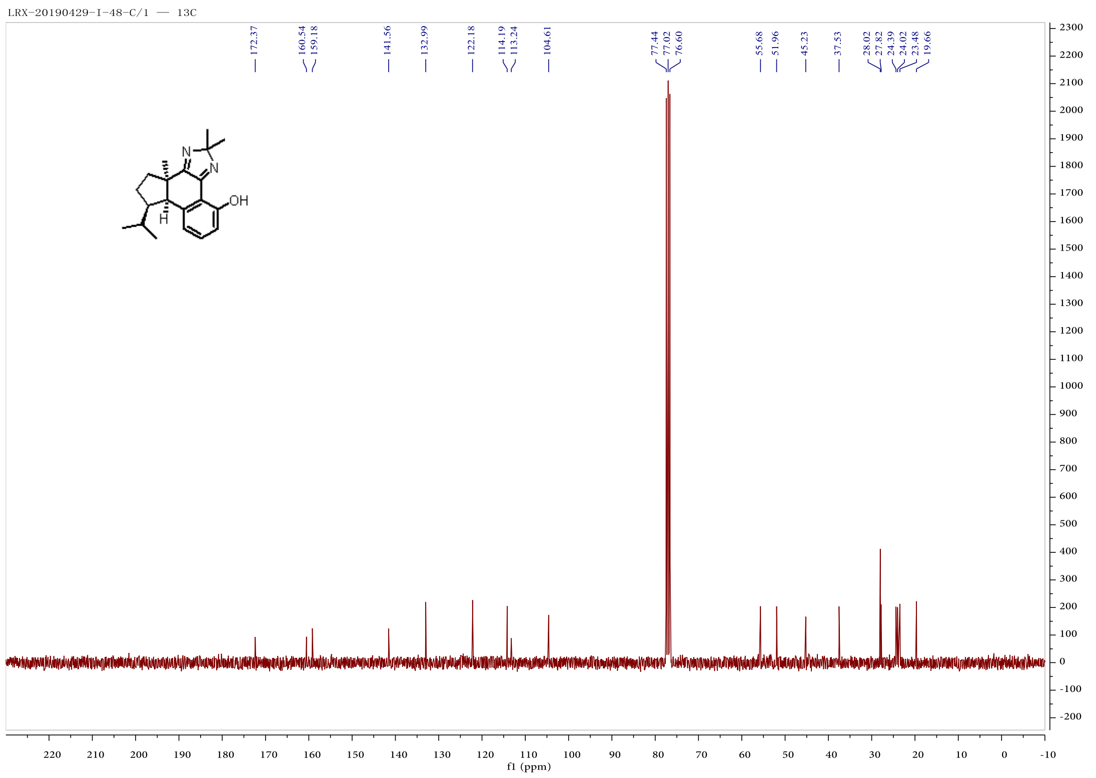
LRX-20190429-I-48-C/1 — 13C
172.37
160.54
159.18
141.56
132.99
122.18
114.19
113.24
104.61
77.44
77.02
76.60
55.68
51.96
45.23
37.53
28.02
27.82
24.39
24.02
23.48
19.66
OH
H
N
N
f1 (ppm)

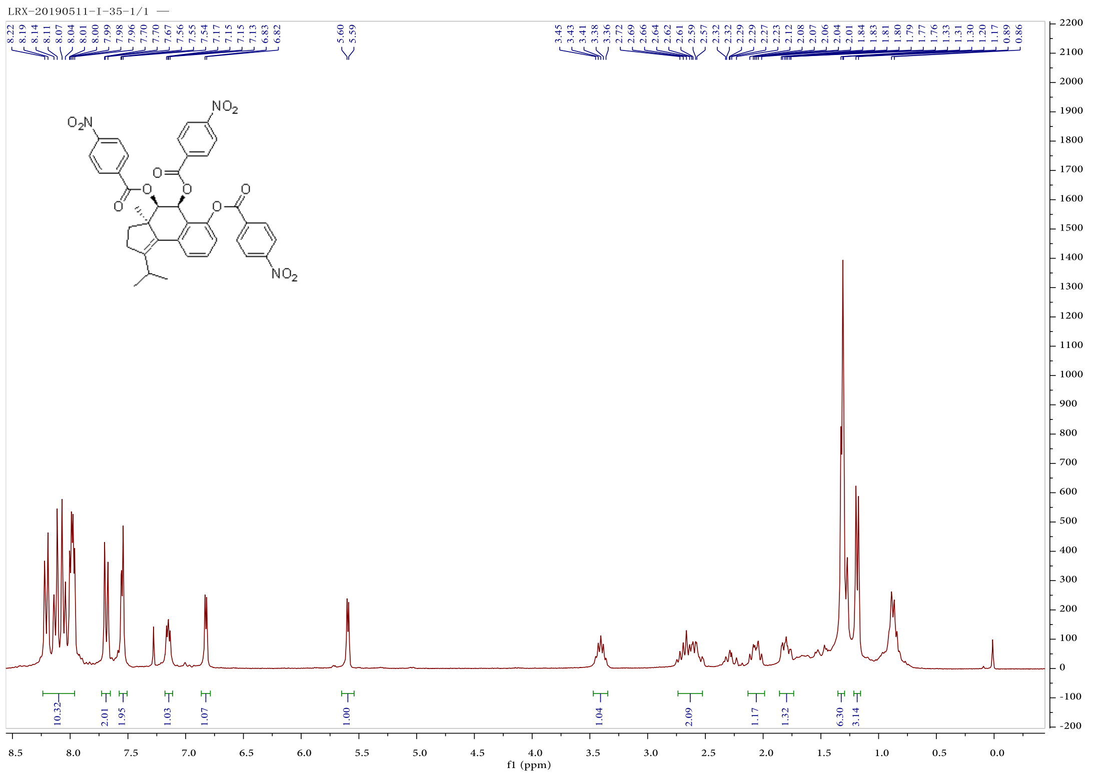
LRX-20190511-I-35-1/1 —
8.22 8.19 8.14 8.11 8.07 8.04 8.01 8.00 7.99 7.98 7.96 7.70 7.70 7.67 7.56 7.55 7.54 7.17 7.15 7.15 7.13 6.83 6.82
5.60 5.59
3.45 3.43 3.41 3.38 3.36 2.72 2.69 2.66 2.64 2.62 2.61 2.59 2.57 2.32 2.32 2.29 2.27 2.23 2.12 2.08 2.07 2.06 2.04 2.01 1.84 1.83 1.81 1.80 1.79 1.77 1.76 1.33 1.31 1.30 1.20 1.17 0.89 0.86
NO_2
O_2N
NO_2
10.32 2.01 1.95 1.03 1.07 1.00 1.04 2.09 1.17 1.32 6.30 3.14
f1 (ppm)

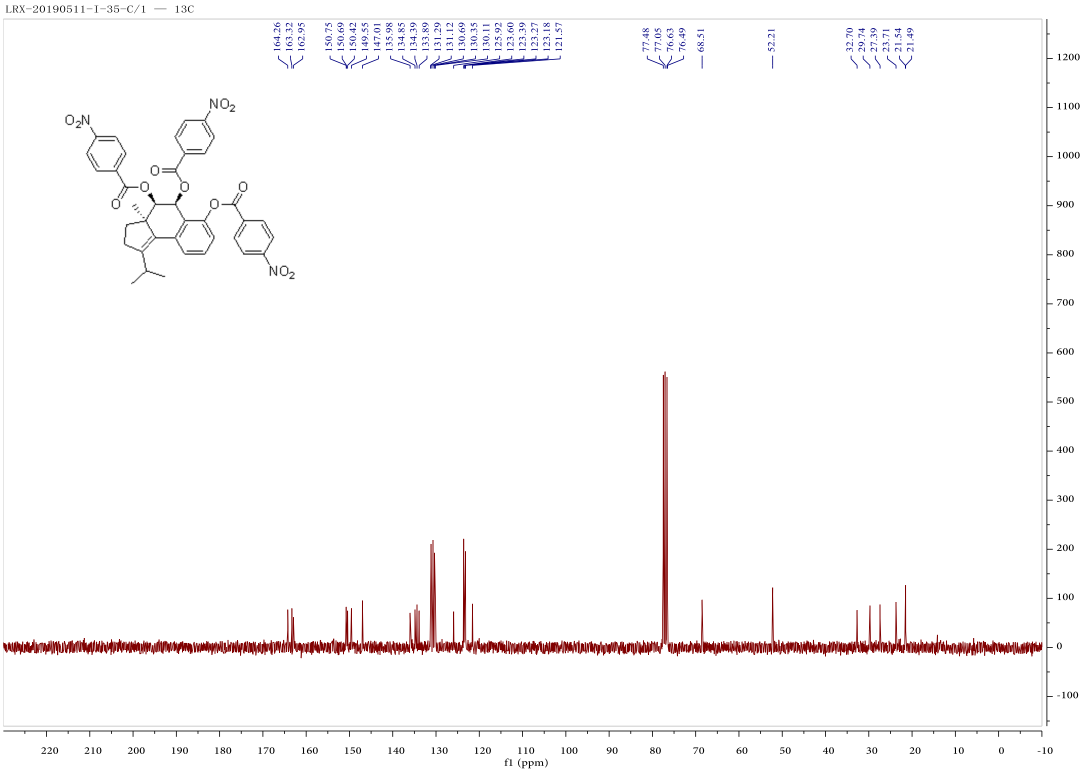
LRX-20190511-I-35-C/1 — 13C
164.26
163.32
162.95
150.75
150.69
150.42
149.55
147.01
135.98
134.85
134.39
133.89
131.29
131.12
130.69
130.35
130.11
125.92
123.60
123.39
123.27
123.18
121.57
77.48
77.05
76.63
76.49
68.51
52.21
32.70
29.74
27.39
23.71
21.54
21.49
O2N
NO2
O
O
O
O
O
O
NO2
f1 (ppm)

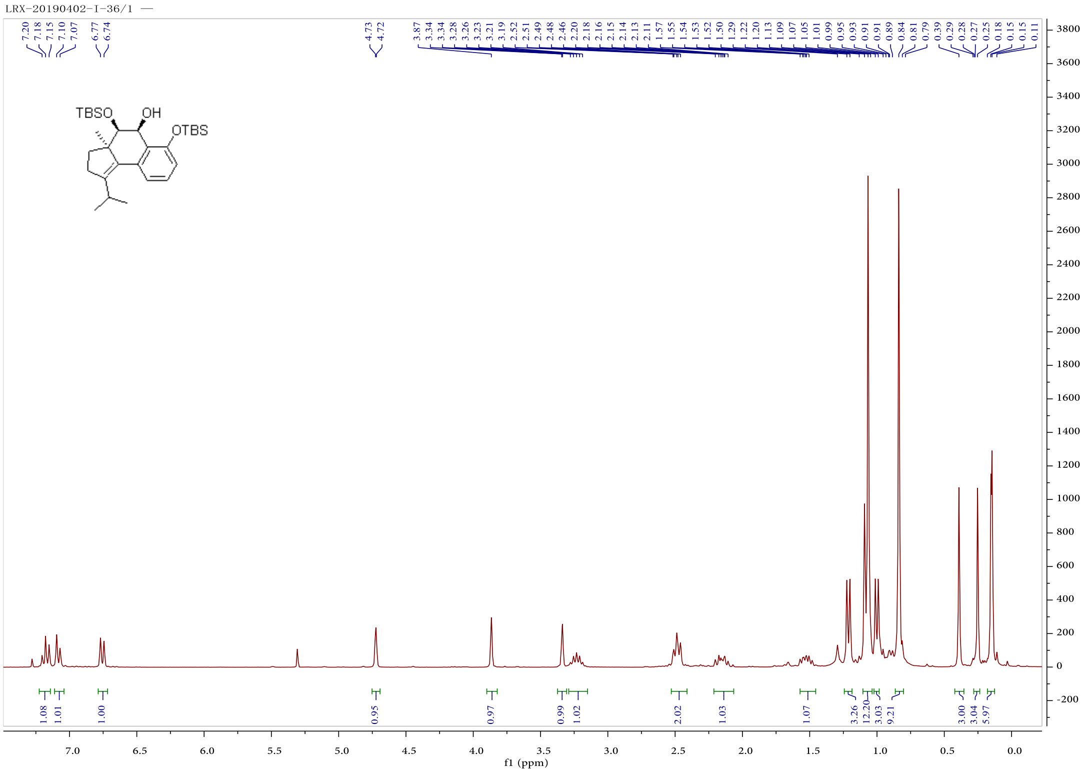
LRX-20190402-I-36/1 —
7.20
7.18
7.15
7.10
7.07
6.77
6.74
4.73
4.72
3.87
3.34
3.34
3.28
3.26
3.23
3.21
3.19
2.52
2.51
2.49
2.48
2.46
2.20
2.18
2.16
2.15
2.14
2.13
2.11
1.57
1.55
1.54
1.53
1.52
1.50
1.29
1.22
1.20
1.13
1.09
1.07
1.05
1.01
0.99
0.95
0.93
0.91
0.91
0.89
0.84
0.81
0.79
0.39
0.29
0.28
0.27
0.25
0.18
0.15
0.11
TBSO
OH
OTBS
1.08
1.01
1.00
0.95
0.97
0.99
1.02
2.02
1.03
1.07
3.26
12.20
3.03
9.21
3.00
3.04
5.97
f1 (ppm)

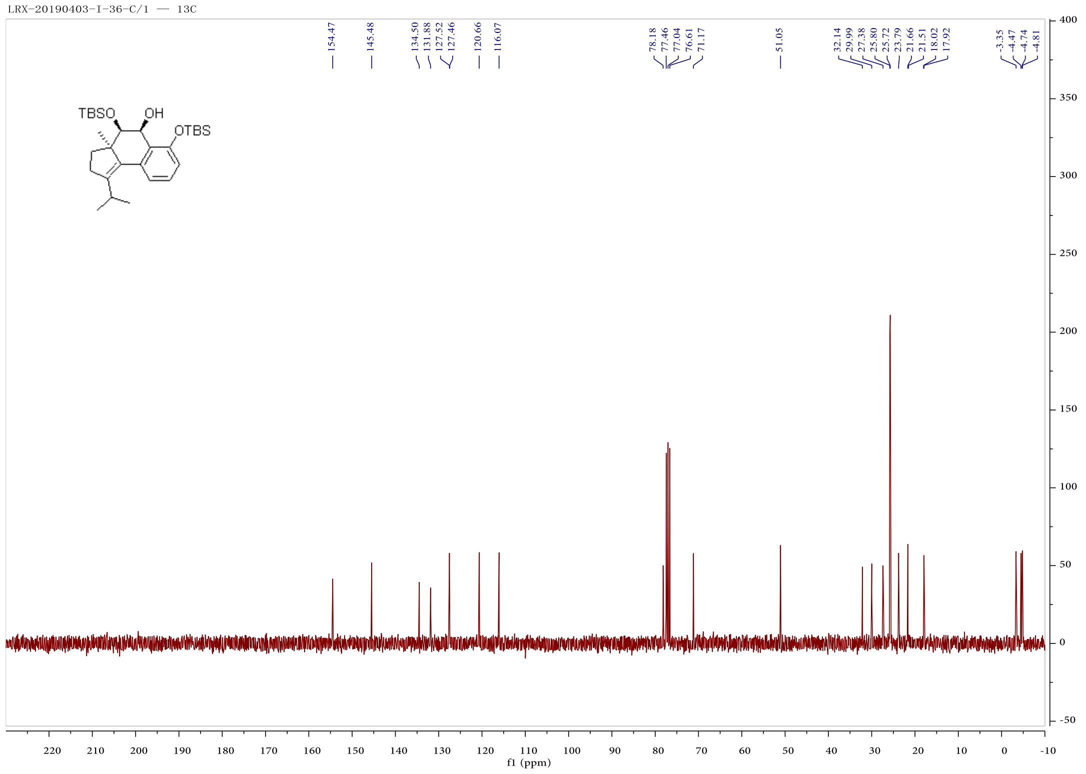
LRX-20190403-I-36-C/1 — 13C
TBSO
OH
OTBS
f1 (ppm)

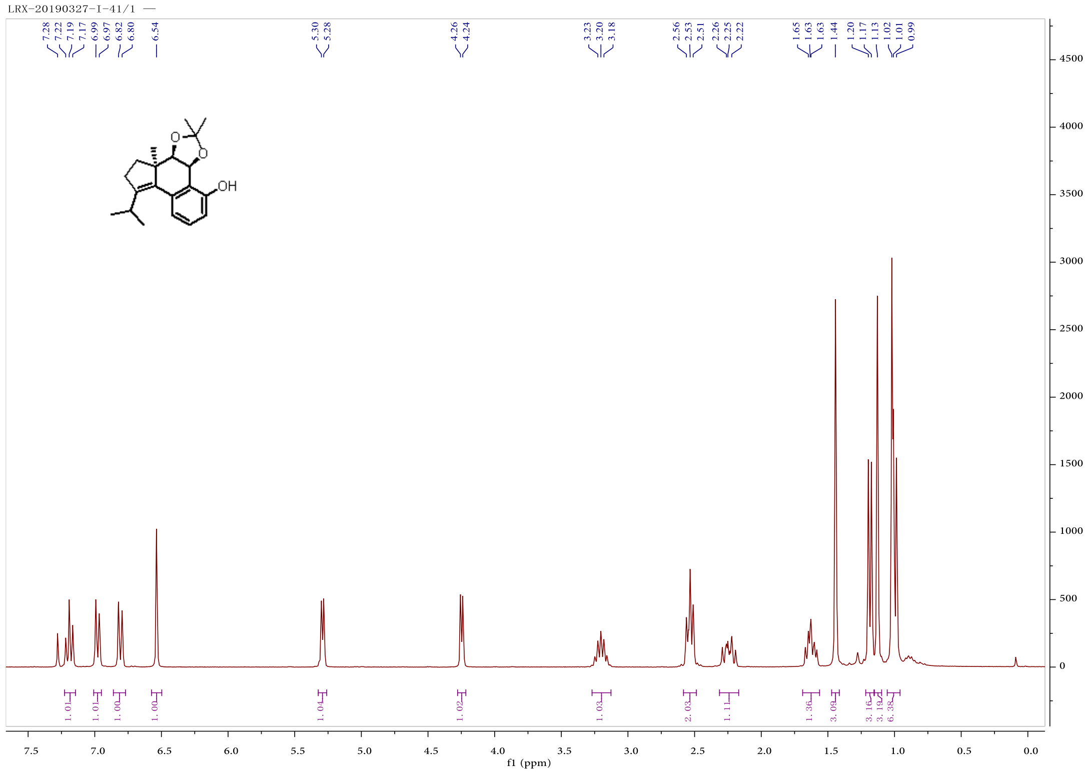
LRX-20190327-I-41/1 —
O
O
OH
f1 (ppm)

LRX-20190327-I-41-C/1 — 13C

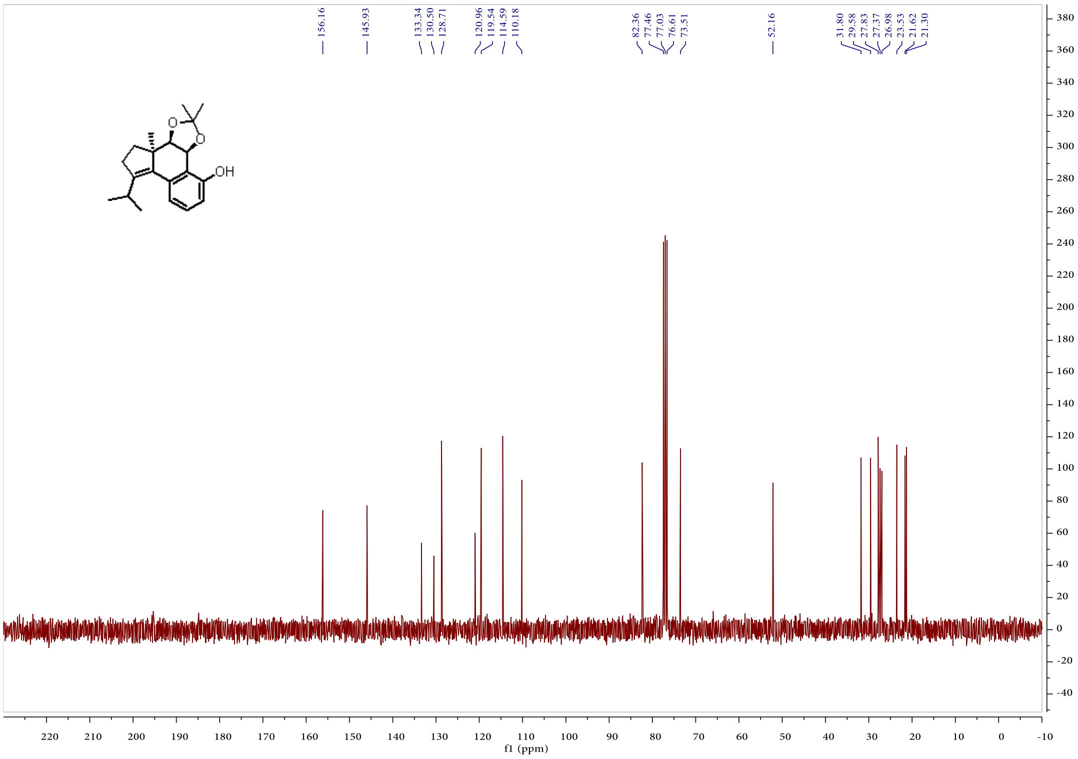

LRX-20190513-I-43-NOE/4 — 1H — sample: 20190509

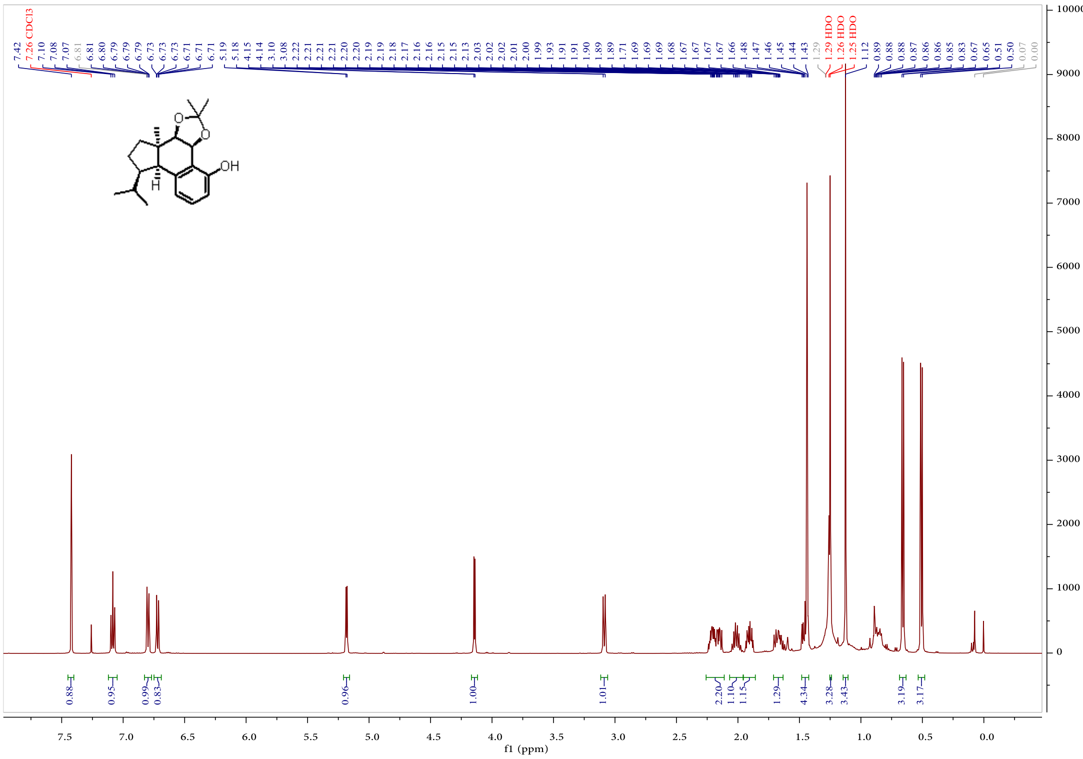

pdata/1 — NOESY — sample: 20190509

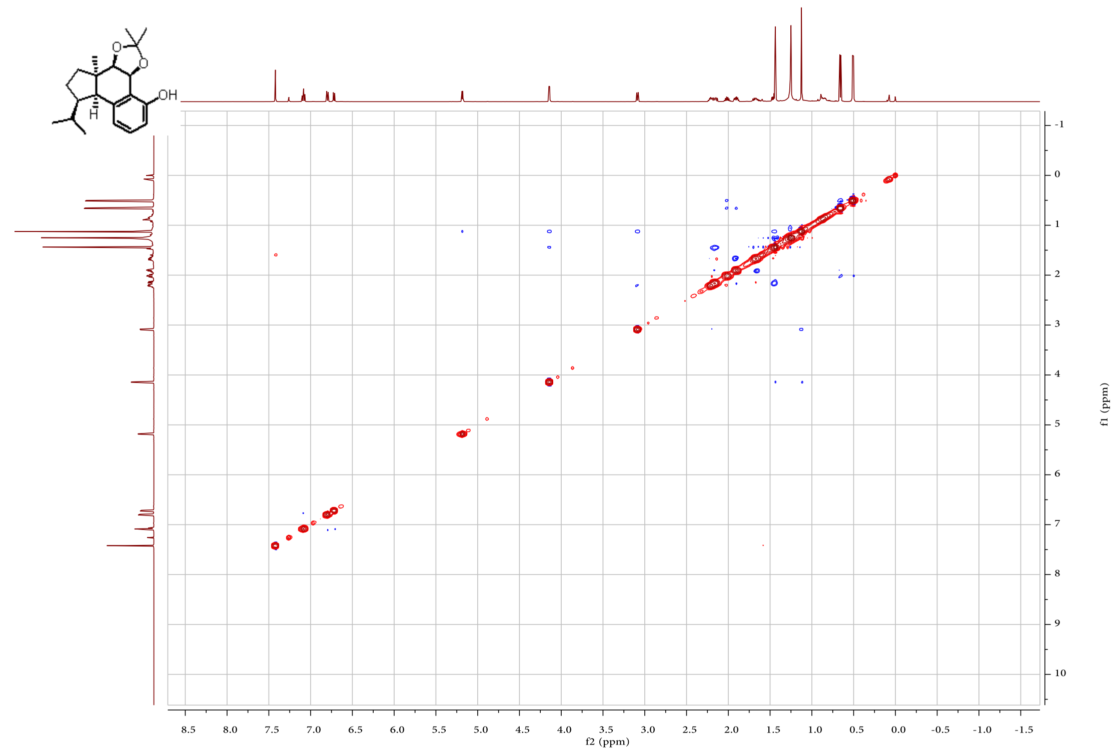

LRX-20190330-I-43-C/1 — 13C

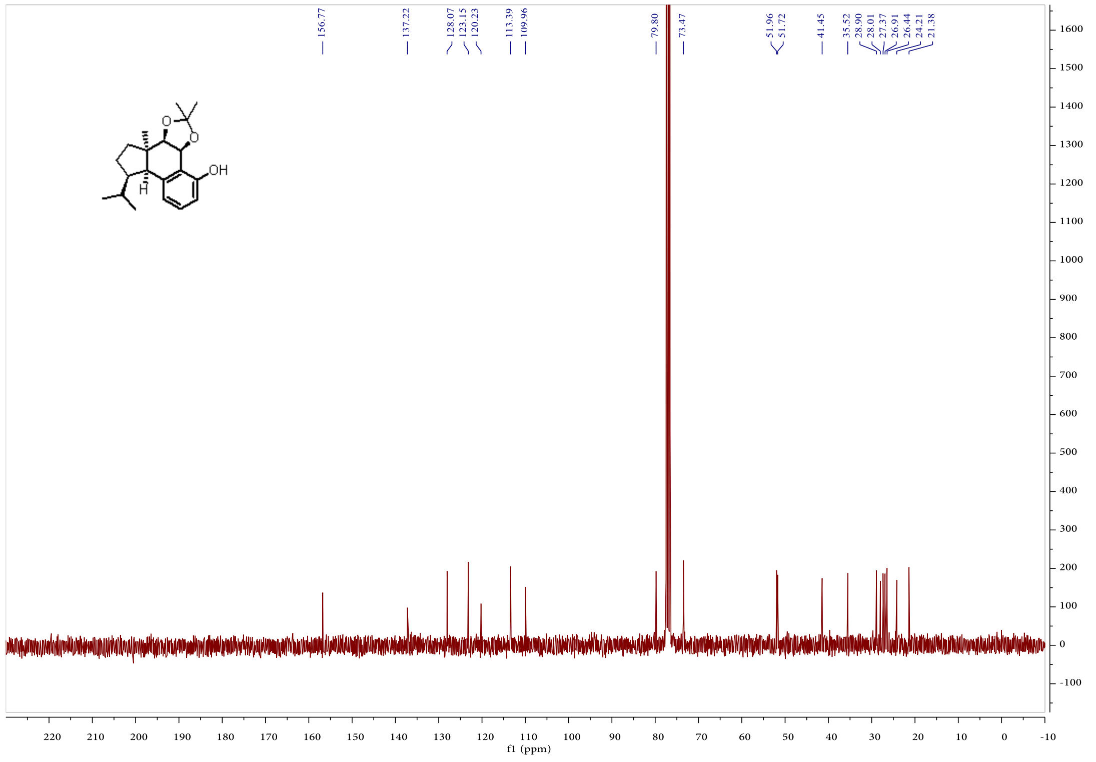

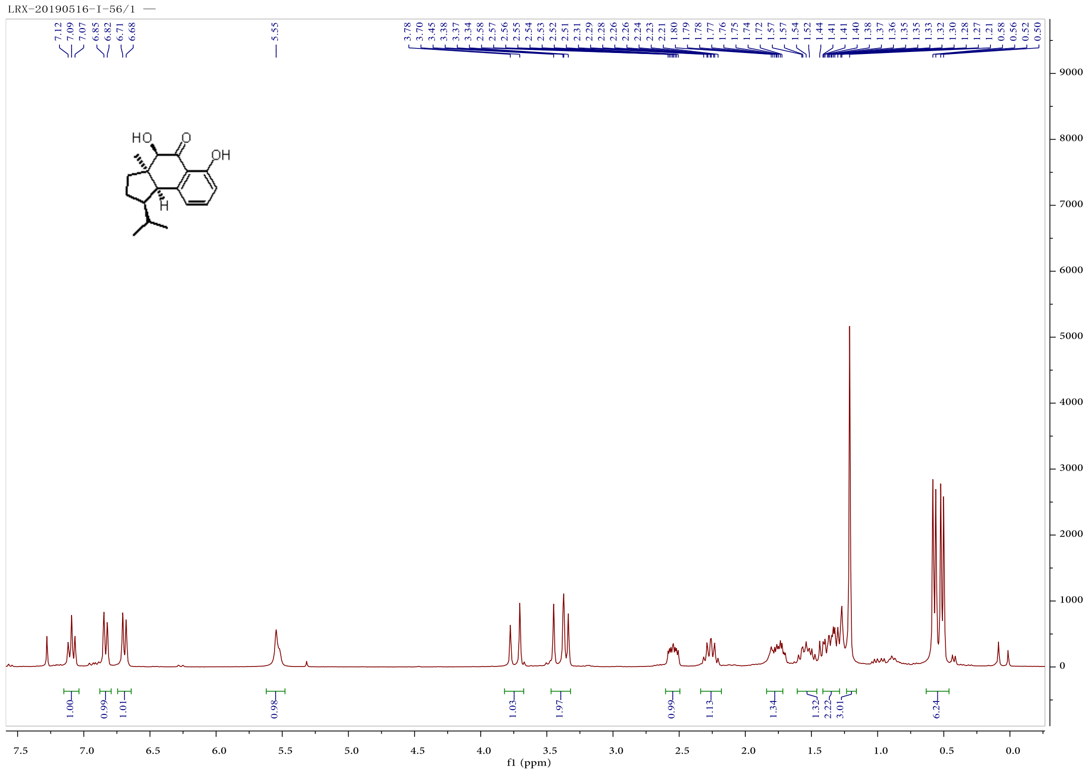
LRX-20190516-I-56/1 —
f1 (ppm)

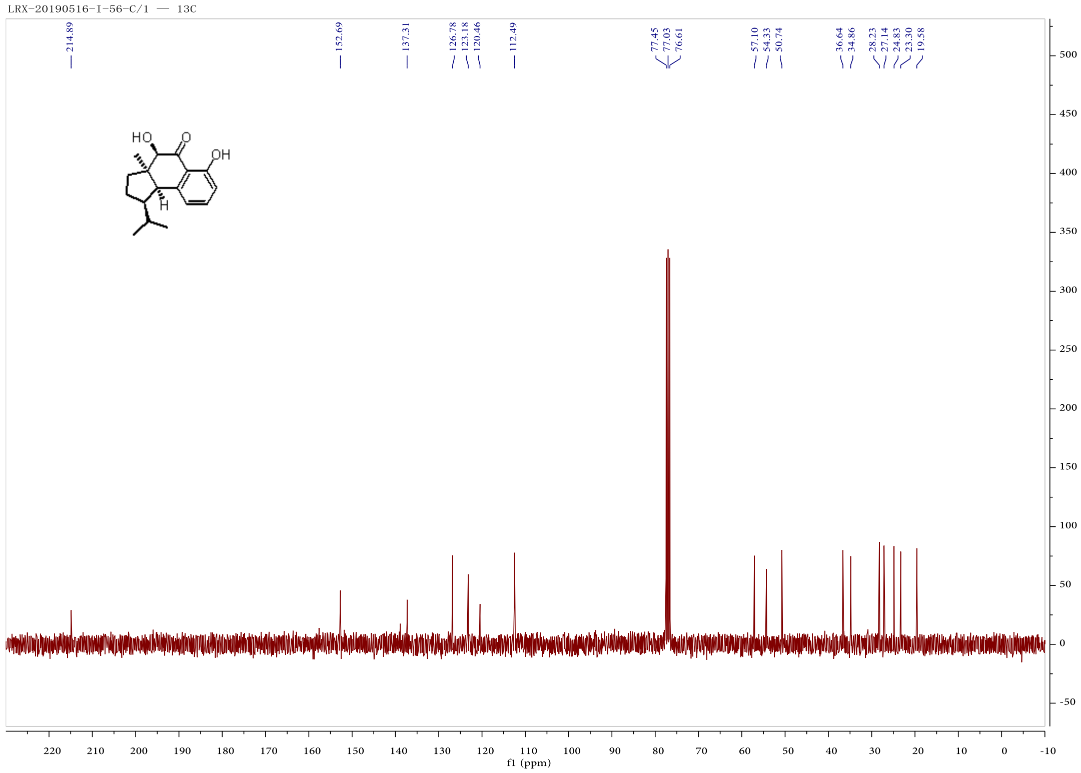
LRX-20190516-I-56-C/1 — 13C
f1 (ppm)

LRX-20190322-I-39/1 —

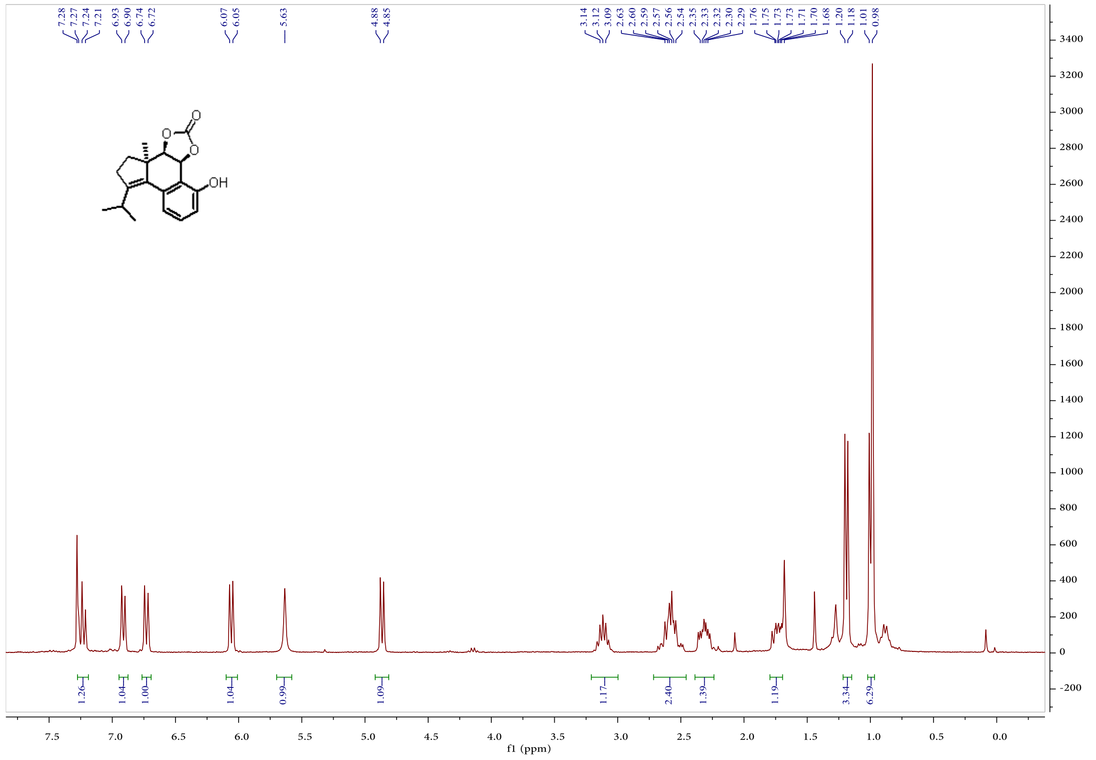

LRX-20190328-I-39-C/1 — 13C

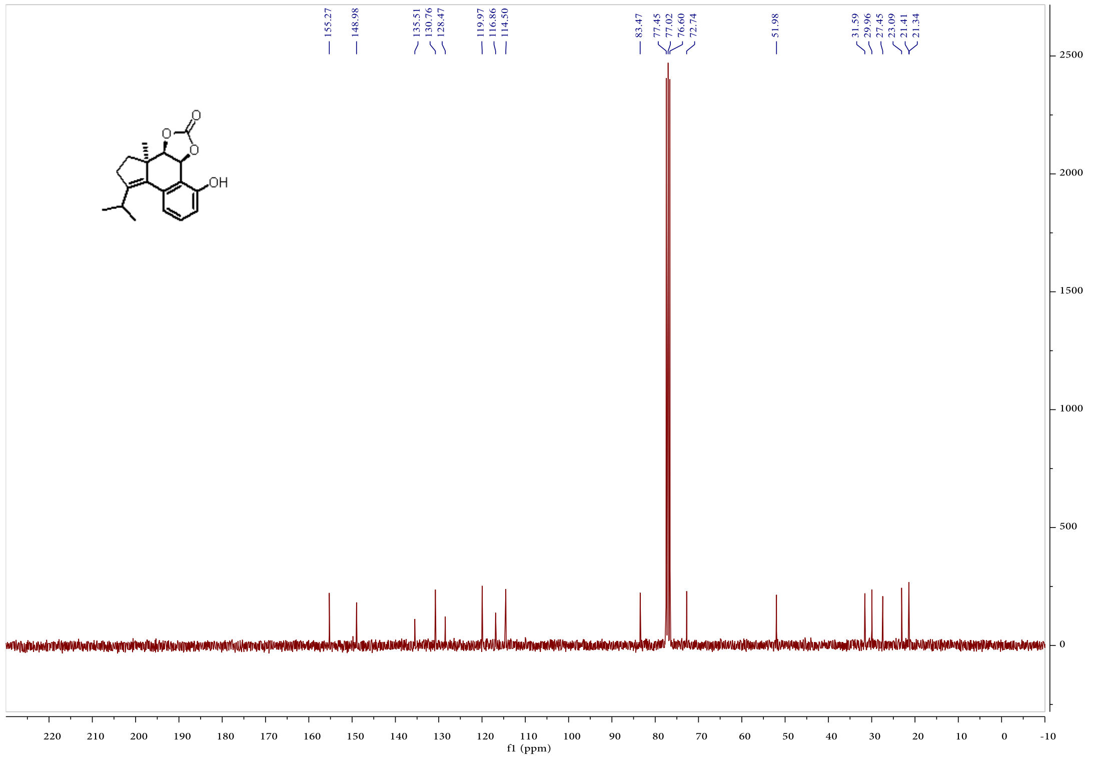

LRX-20190510-I-45/1 —

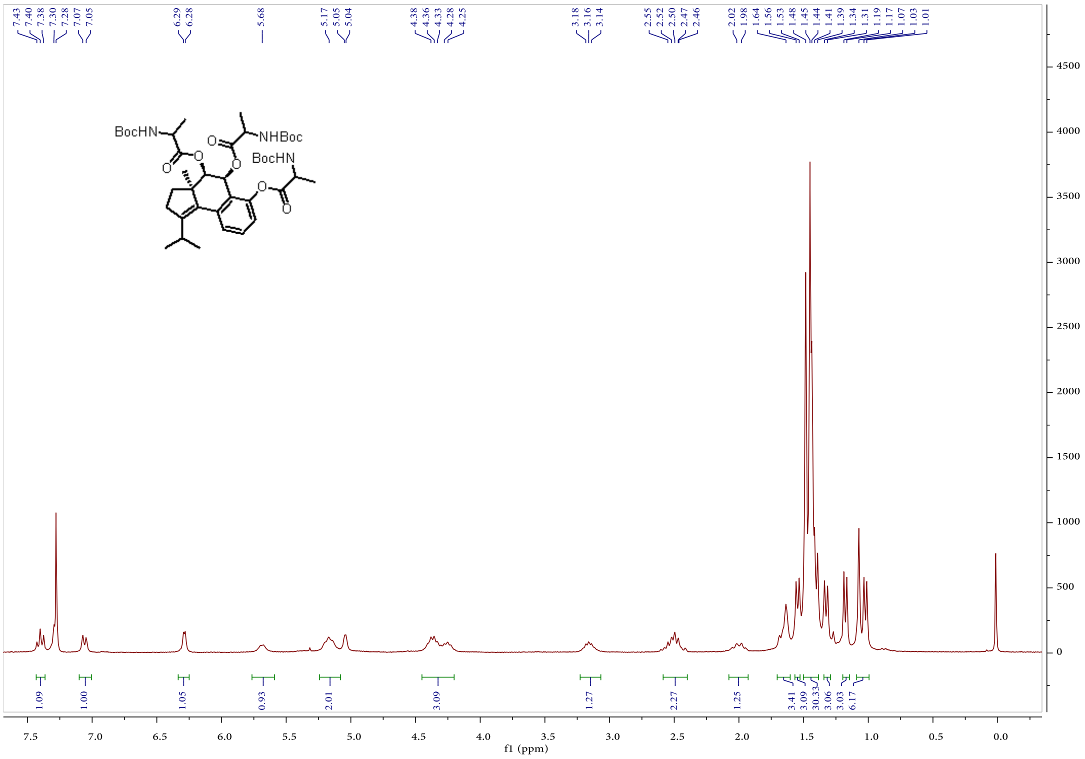

LRX-20190509-I-45-1-C/1 — 13C

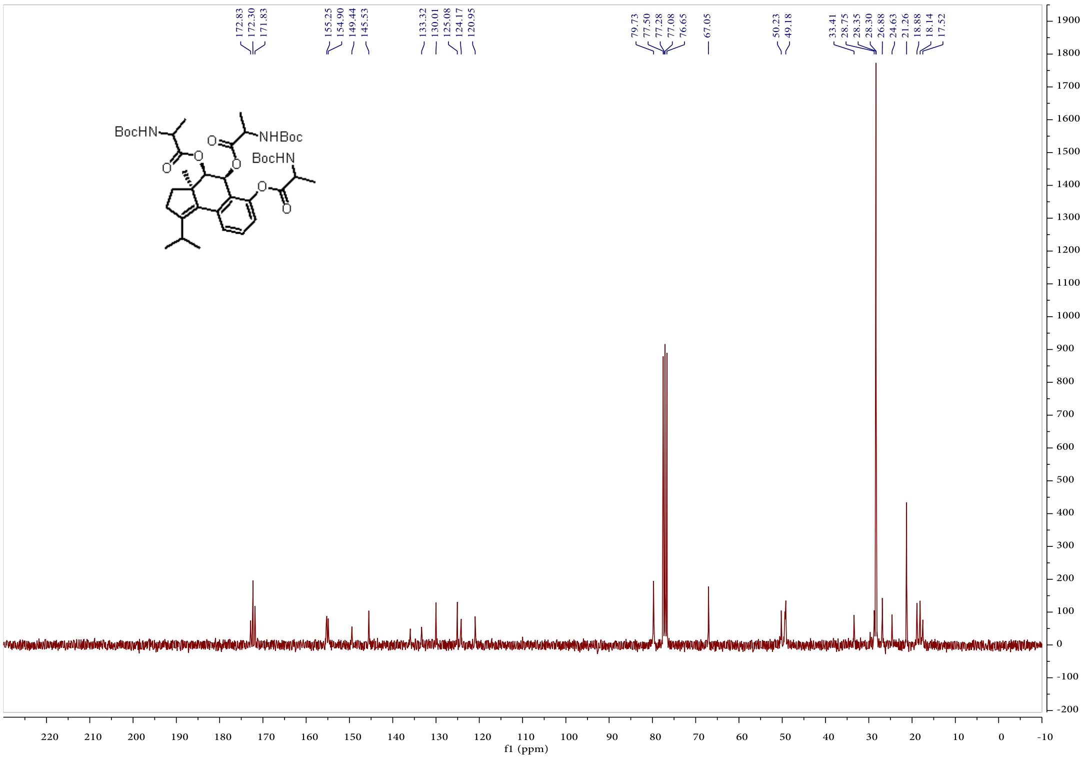

LRX-20190410-I-46/1 —

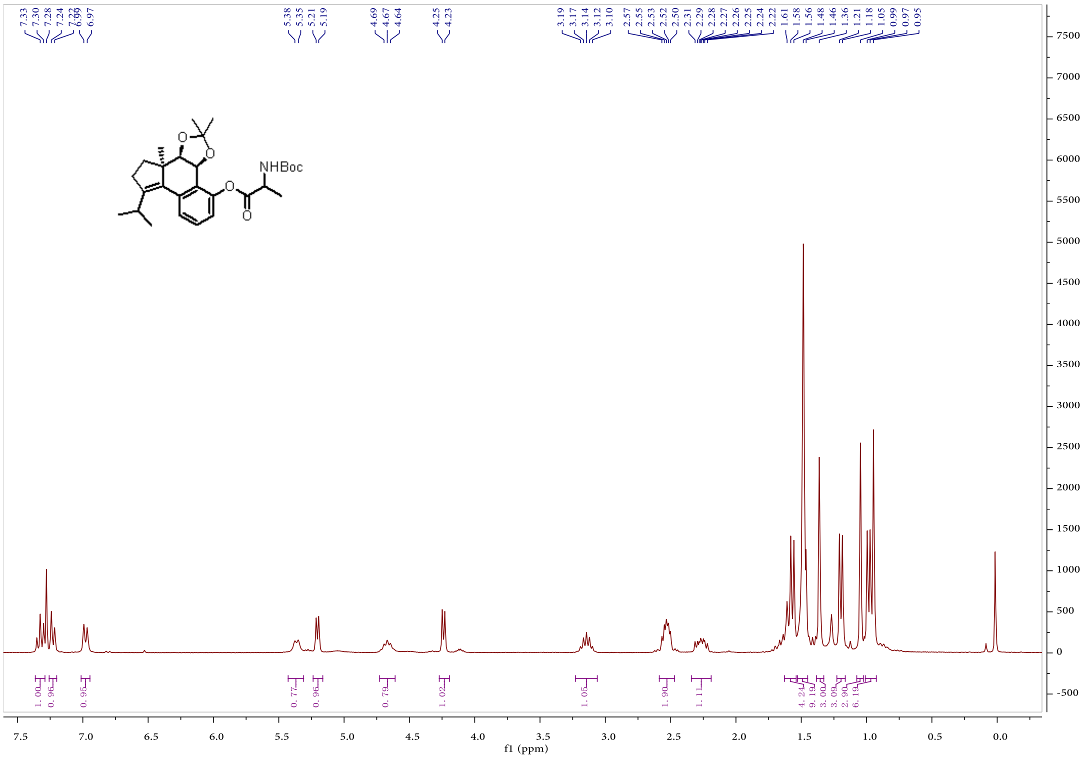

LRX-20190418-I-46-C/1 — 13C

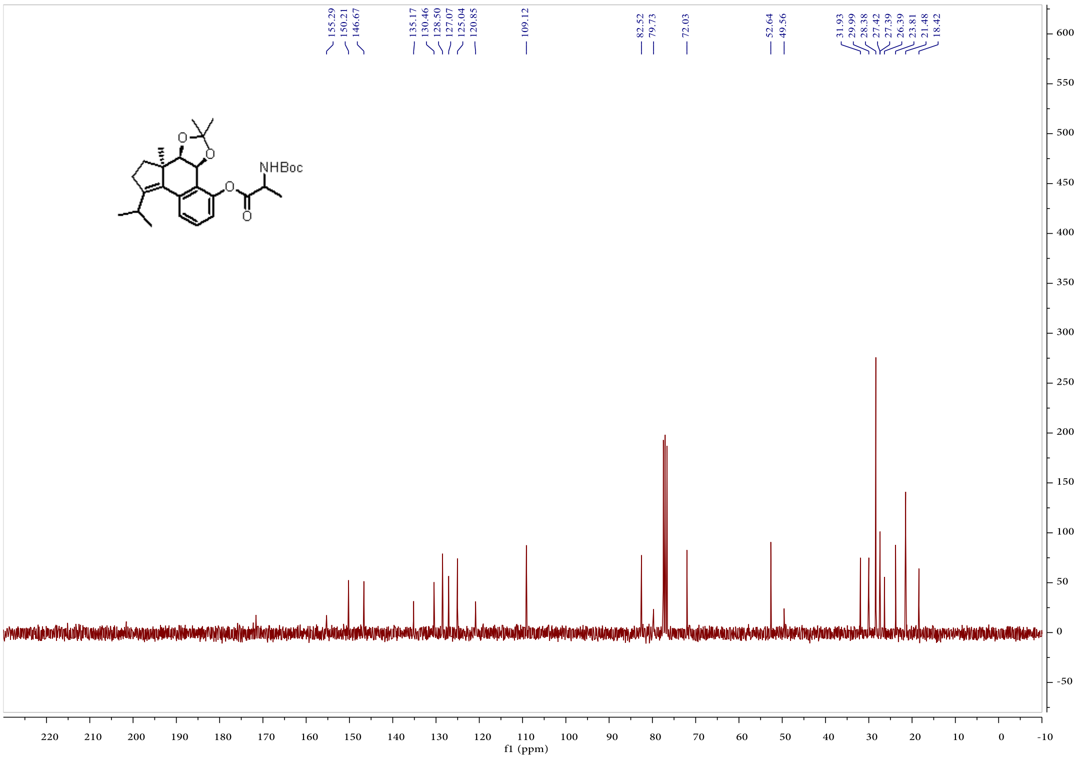

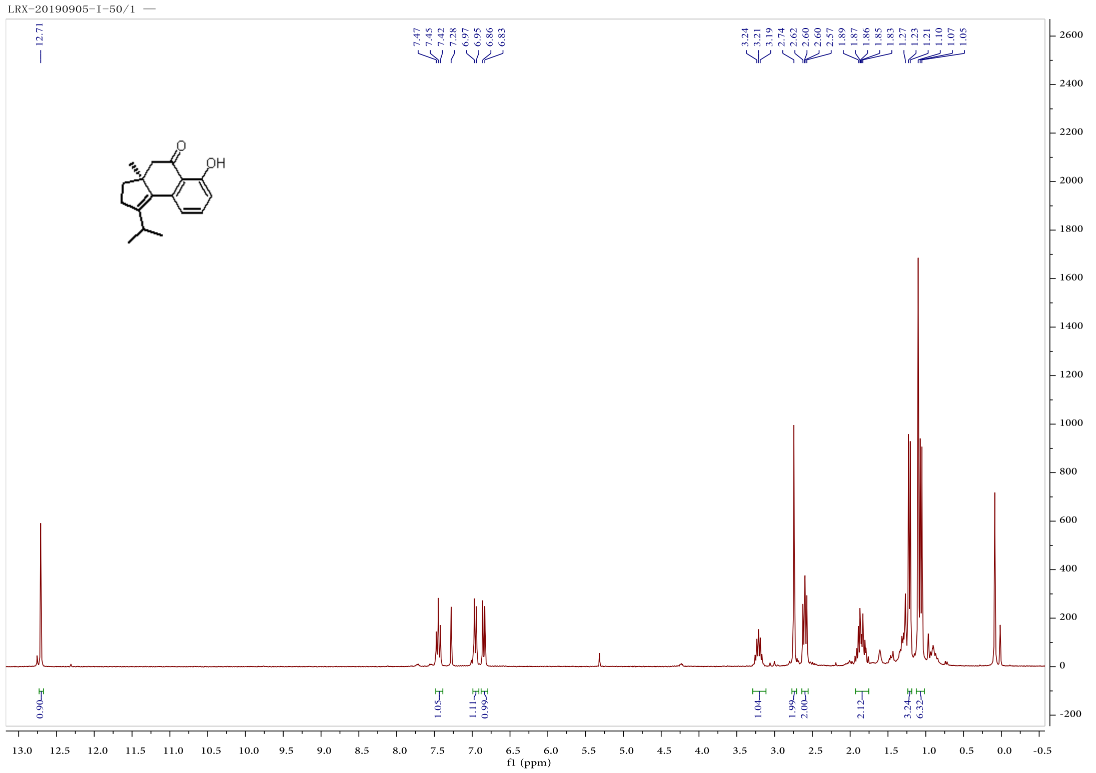
LRX-20190905-I-50/1 —
12.71
7.47 7.45 7.42 7.28 6.97 6.95 6.86 6.83
3.24 3.21 3.19 2.74 2.62 2.60 2.60 2.57 1.89 1.87 1.86 1.85 1.83 1.27 1.23 1.21 1.10 1.07 1.05
OH
0.90 1.05 1.11 0.99 1.04 1.99 2.00 2.12 3.24 6.32
f1 (ppm)

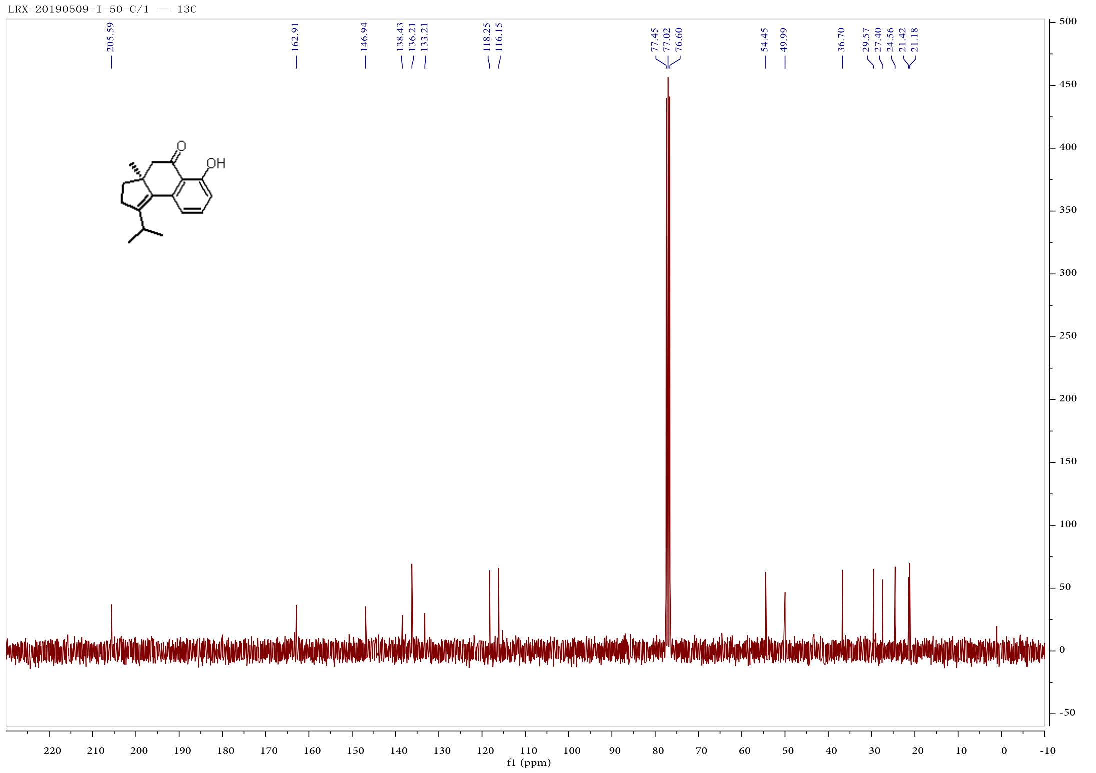
LRX-20190509-I-50-C/1 — 13C
205.59
162.91
146.94
138.43 136.21 133.21
118.25 116.15
77.45 77.02 76.60
54.45 49.99
36.70 29.57 27.40 24.56 21.42 21.18
OH
f1 (ppm)

LRX-20190705-I-67/1 —

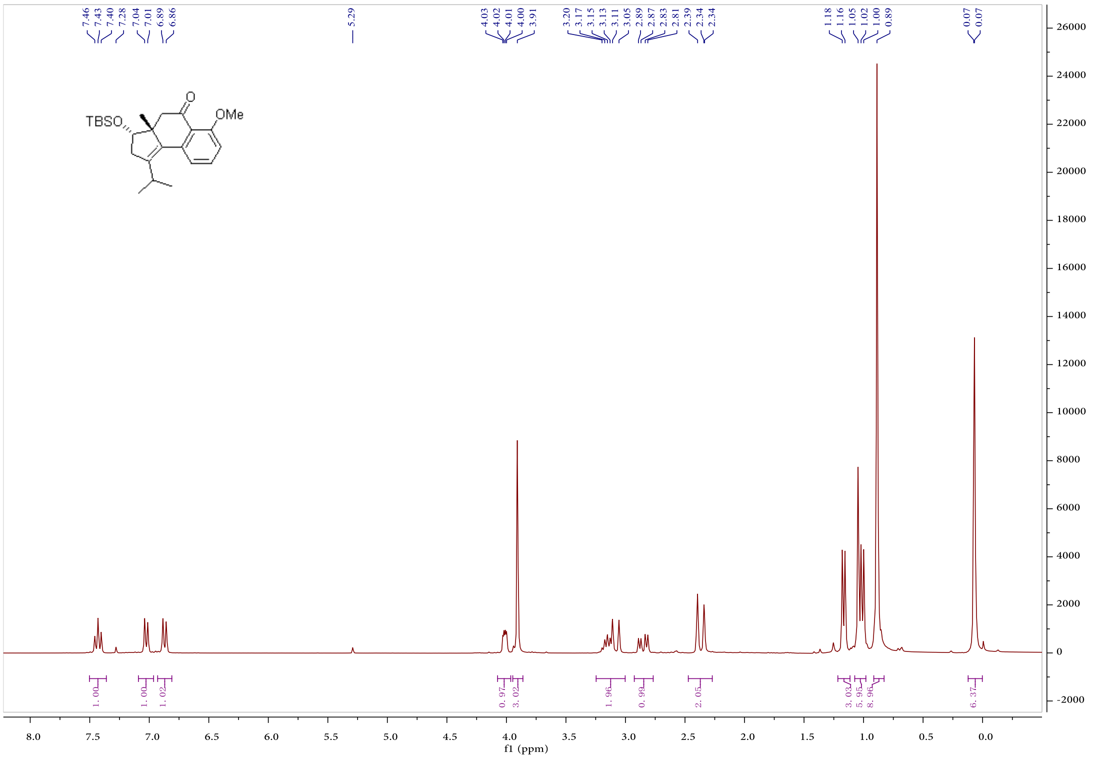

LRX-20190705-I-67-C/1 — 13C

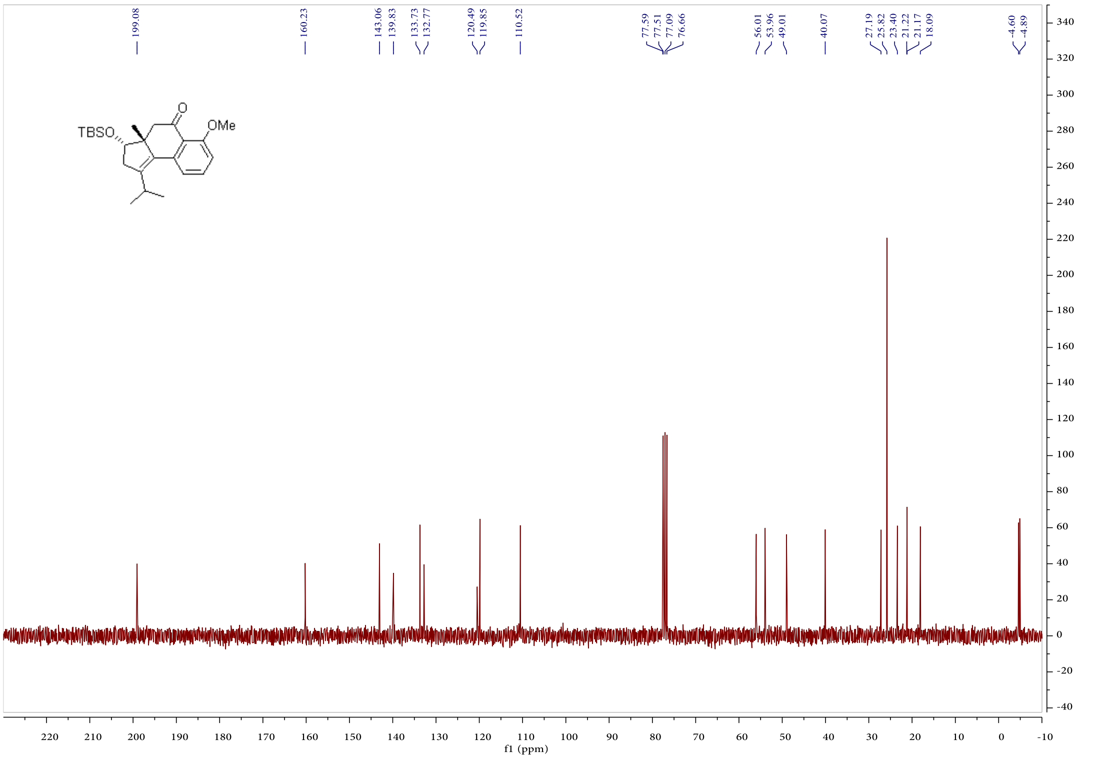

LRX-20190709-I-69/1 —

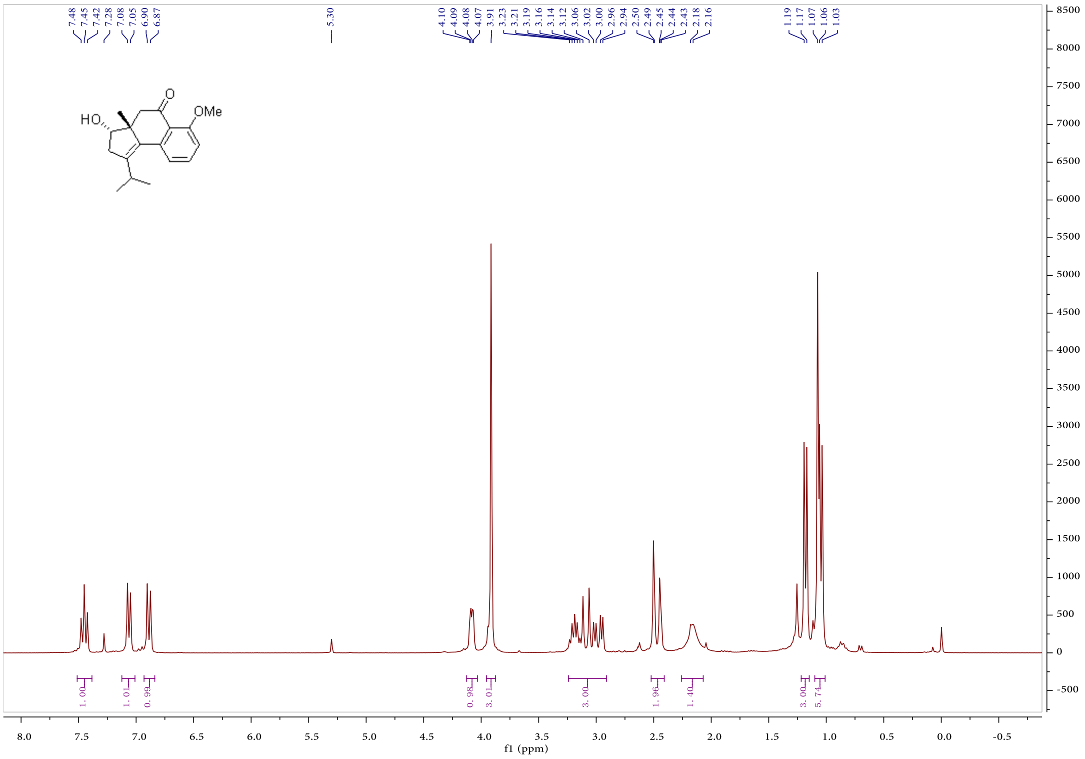

LRX-20190709-I-69-C/1 — 13C

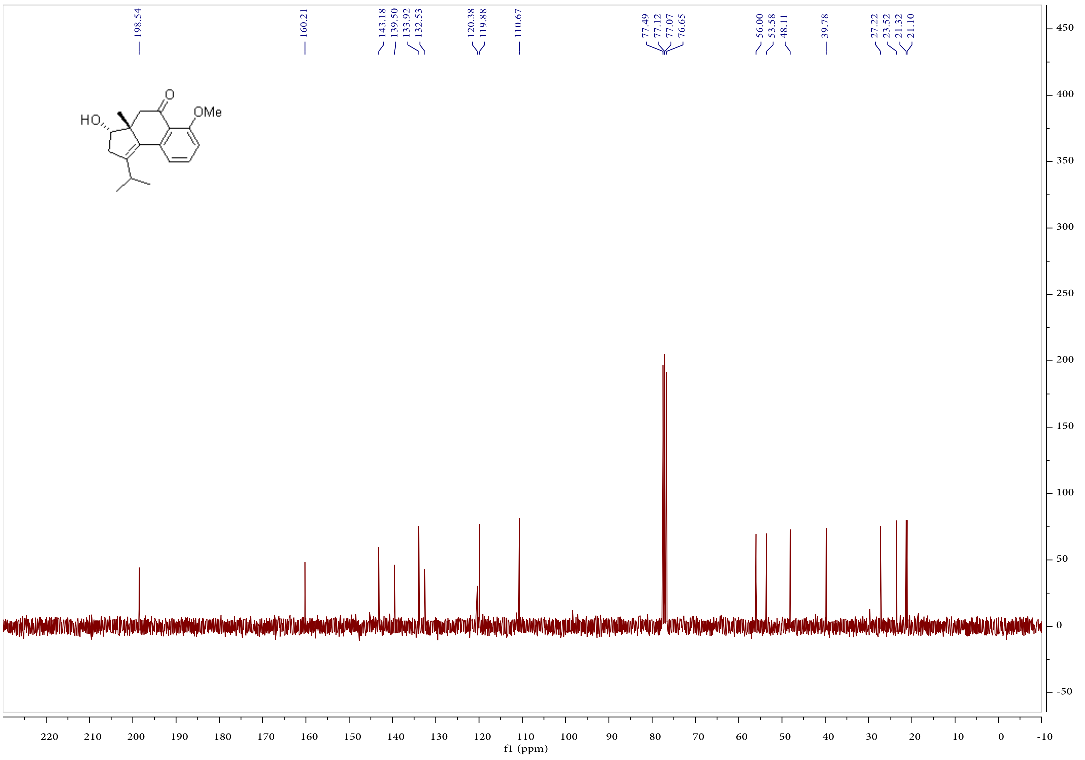

LRX-20190821-I-71/1 —

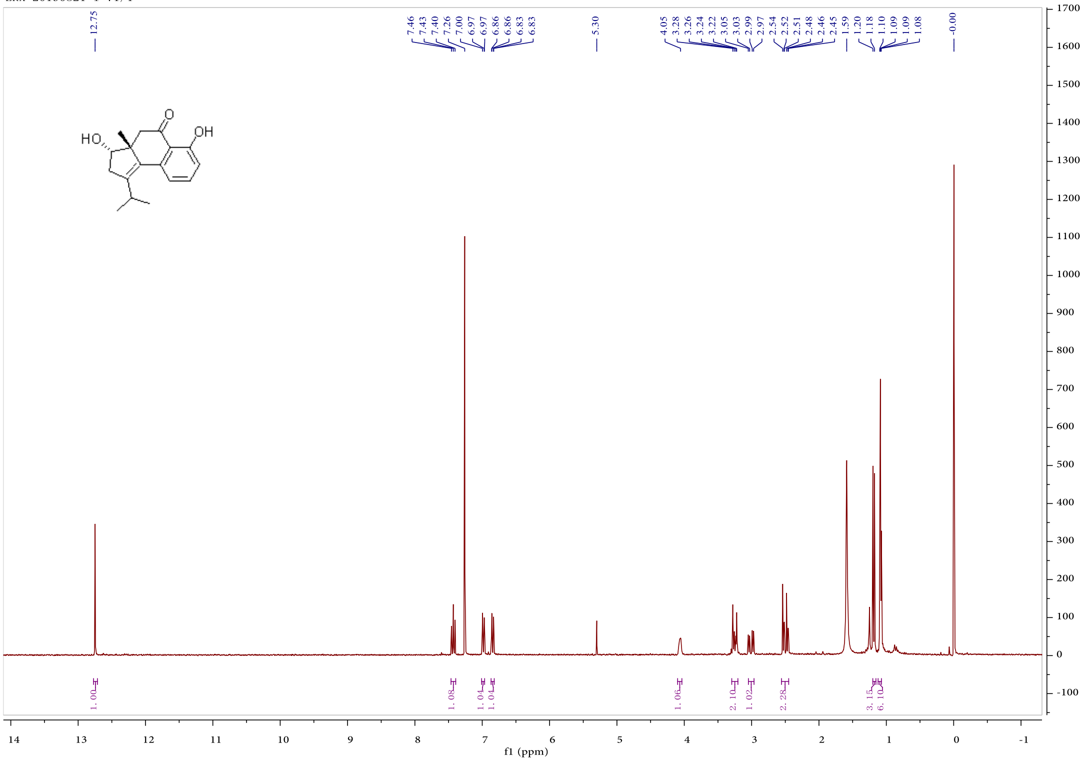

LRX-20190820-I-71-C/1 — 13C

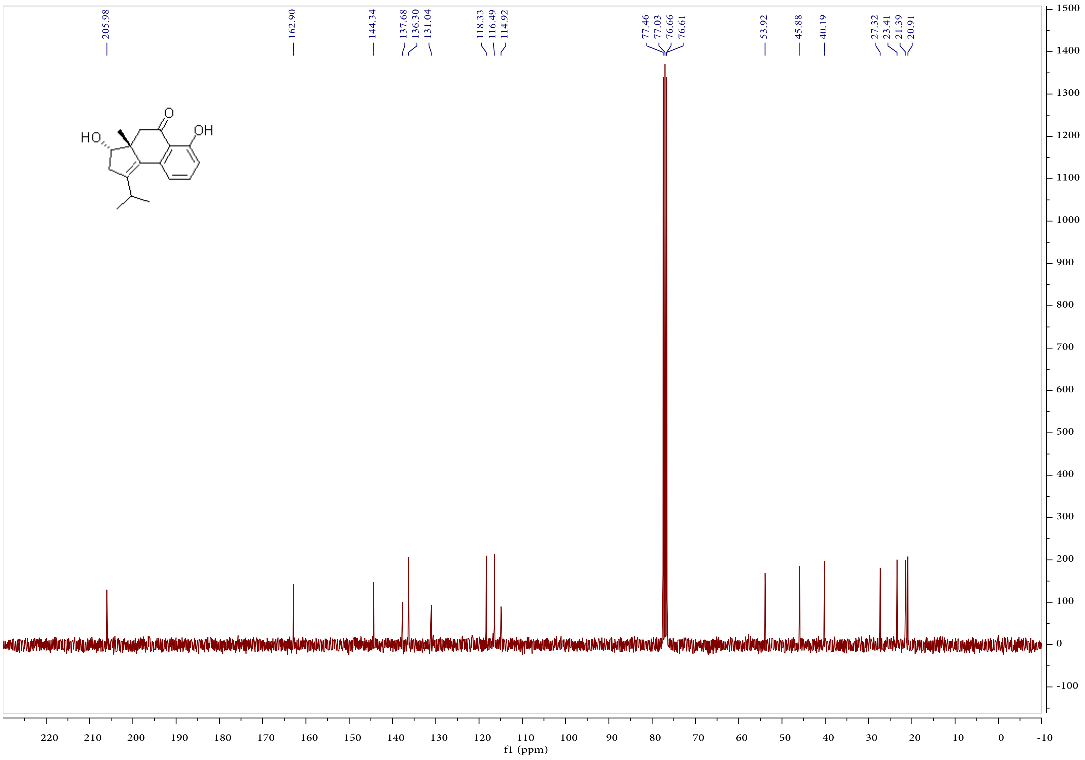

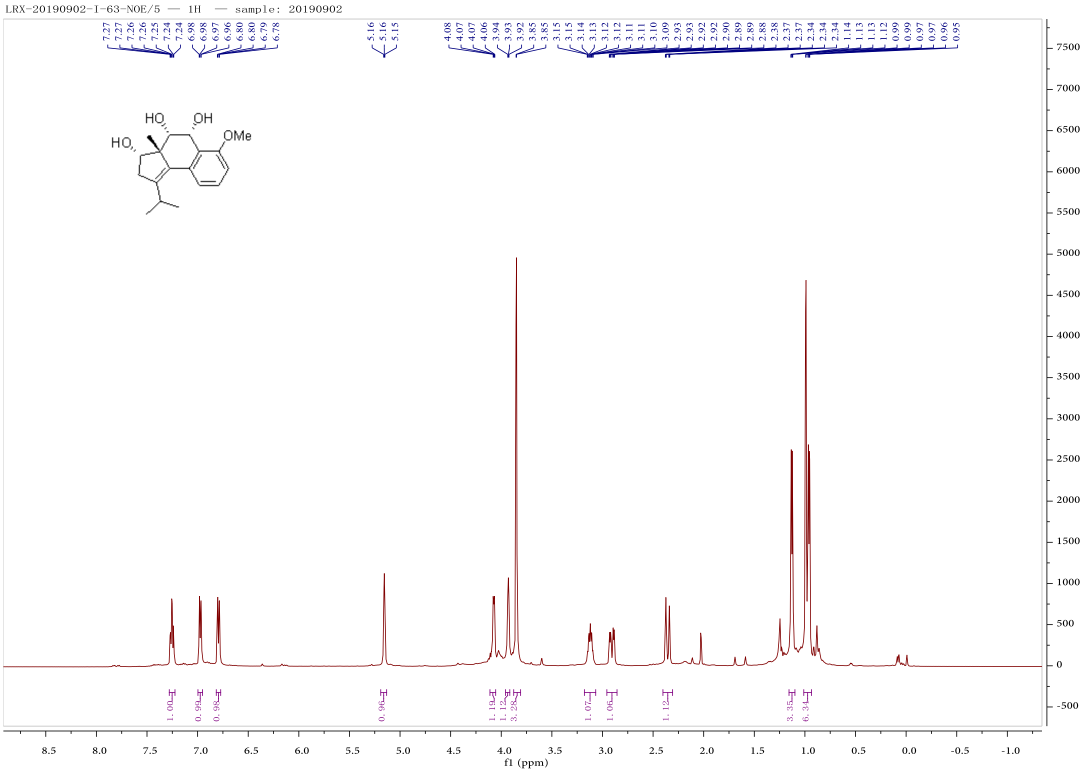
LRX-20190902-I-63-NOE/5 — 1H — sample: 20190902
7.27 7.27 7.26 7.25 7.24 7.24 6.98 6.98 6.97 6.96 6.80 6.80 6.79 6.78 5.16 5.16 5.15 4.08 4.07 4.07 4.06 3.94 3.93 3.92 3.85 3.85 3.15 3.15 3.14 3.13 3.12 3.12 3.11 3.11 3.10 3.09 2.93 2.93 2.92 2.92 2.90 2.89 2.89 2.88 2.38 2.37 2.37 2.34 2.34 2.34 1.14 1.13 1.13 1.13 1.12 0.99 0.99 0.97 0.97 0.96 0.95
HO
OH
HO
OMe
1.00 0.99 0.98 0.96 1.19 1.12 3.28 1.07 1.06 1.12 3.35 6.31
f1 (ppm)

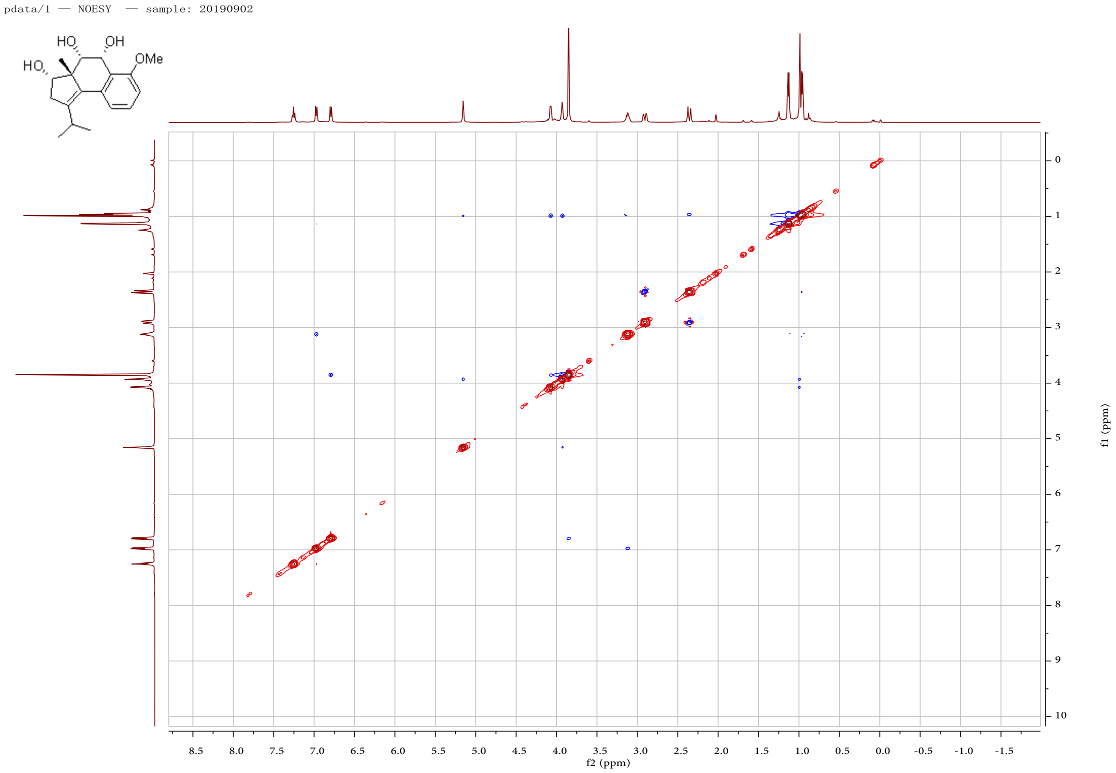
pdata/1 — NOESY — sample: 20190902
HO
OH
HO
OMe
f2 (ppm)
f1 (ppm)

LRX-20190902-I-63-C/1 — 13C

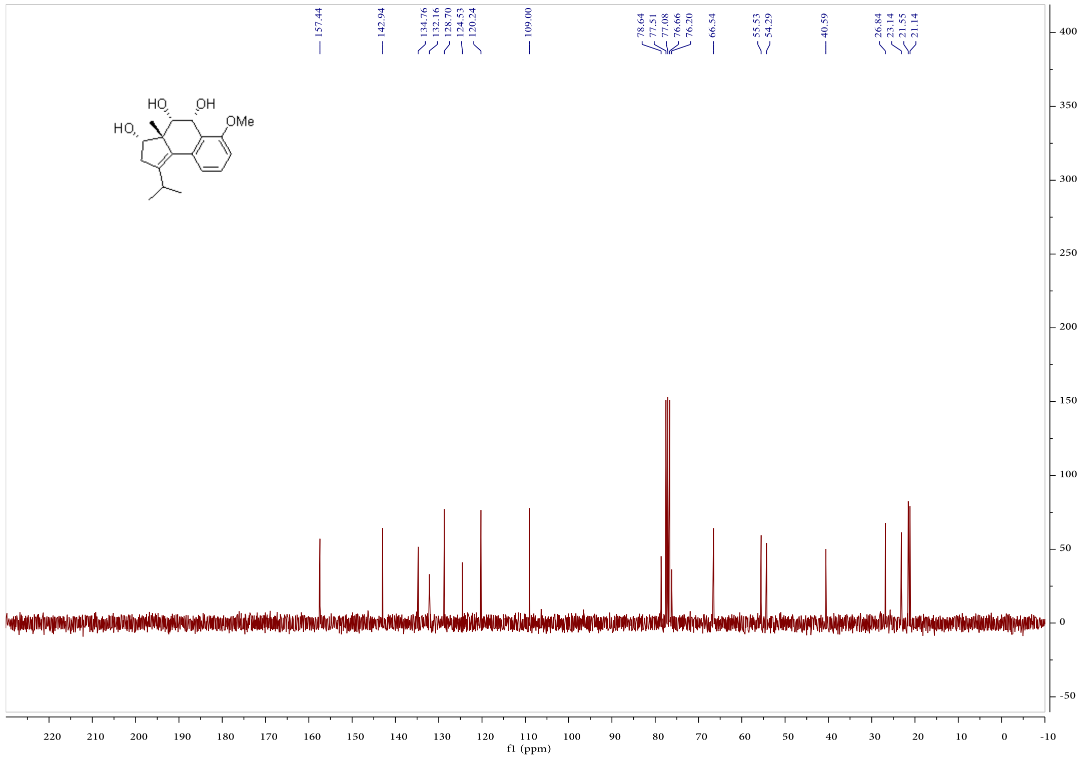

LRX-20190716-I-65/1 —

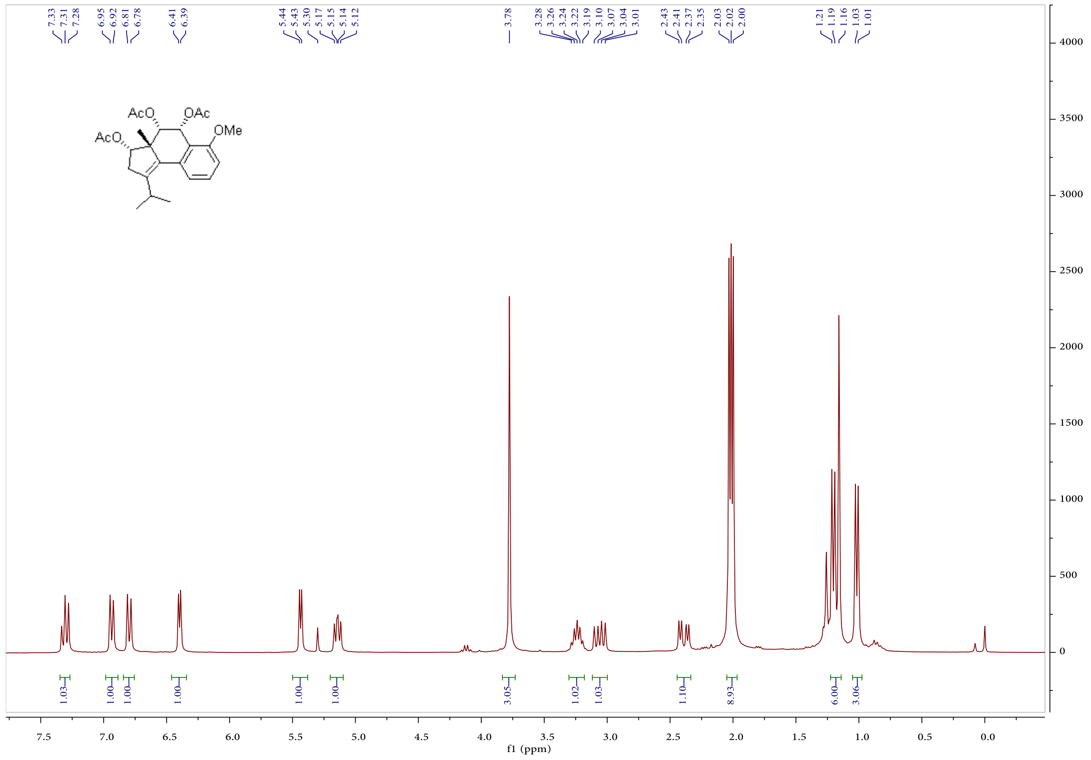

LRX-20190716-I-65-C/1 — 13C

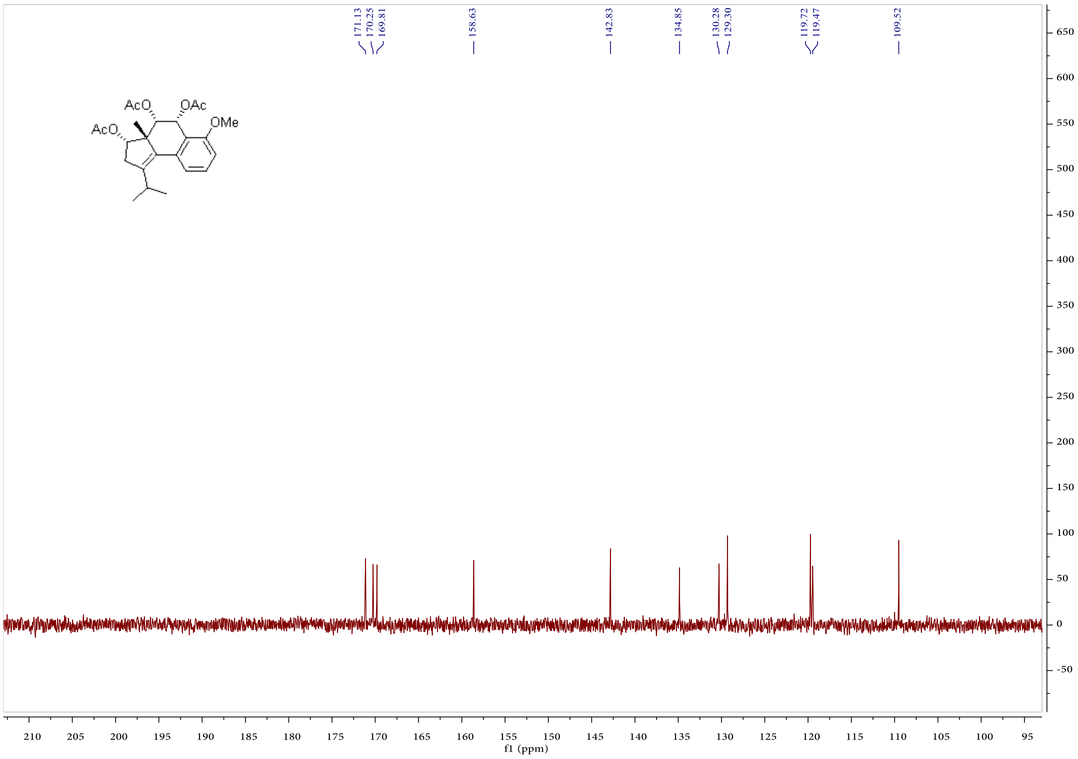

LRX-20190723-I-73/1 —

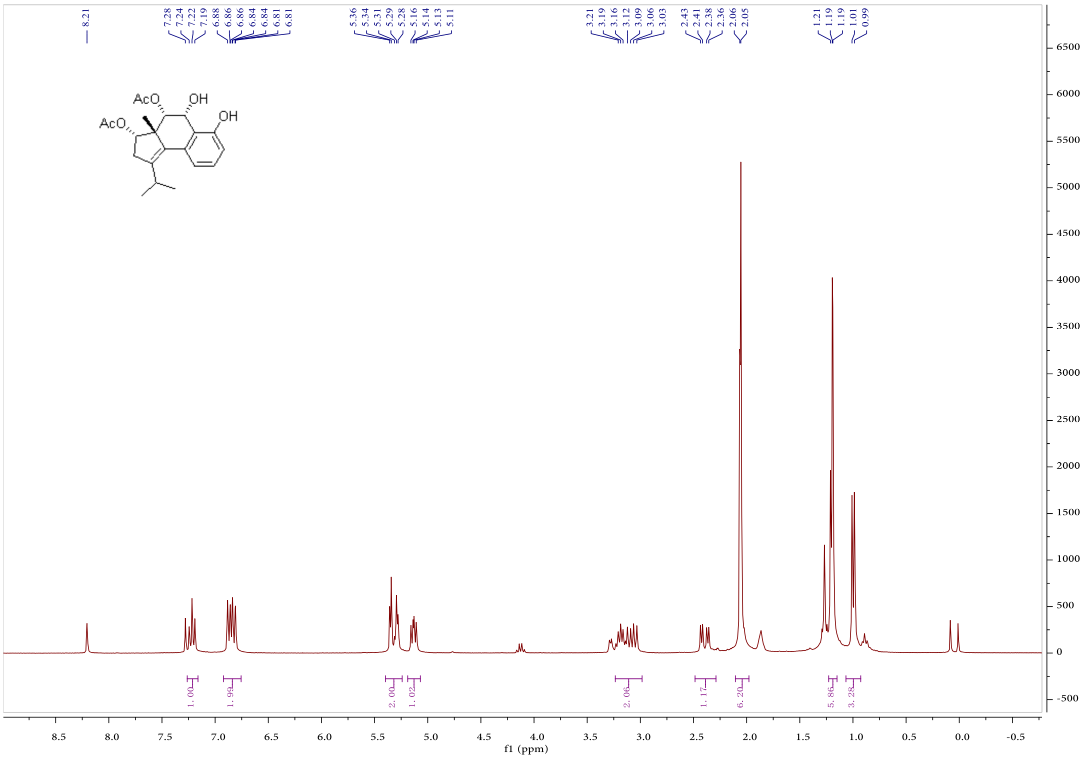

LRX-20190723-I-73-C/1 — 13C

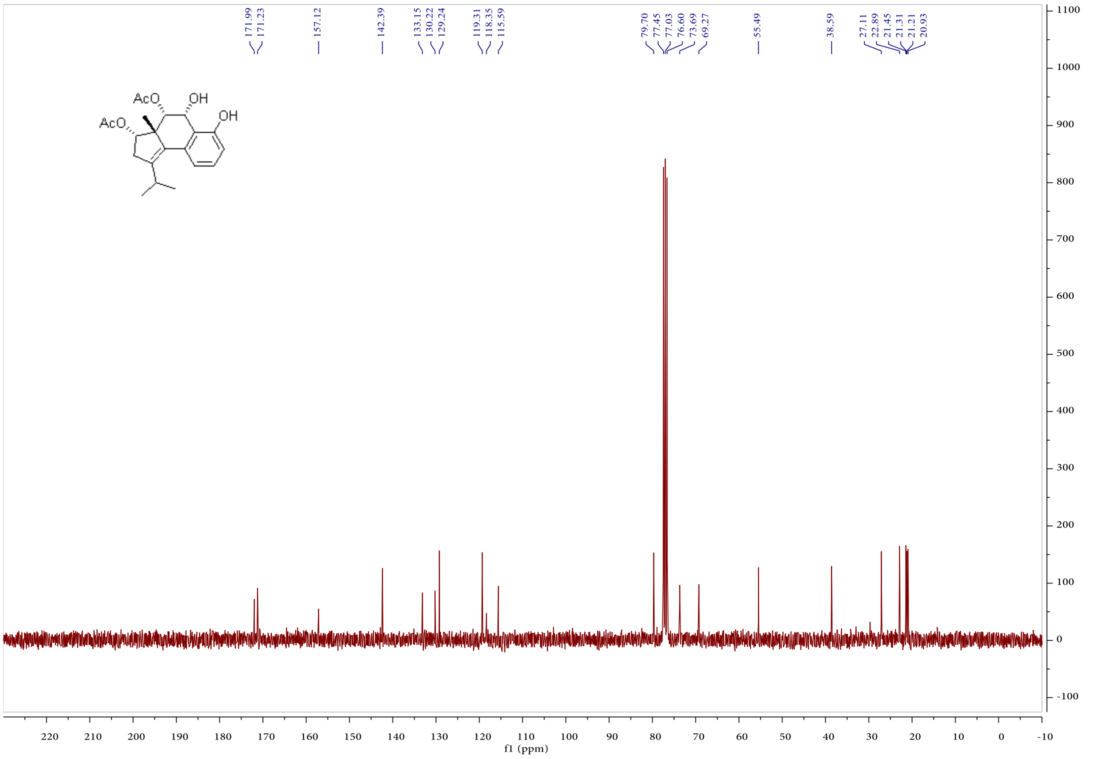

LRX-20190723-I-75/1 —

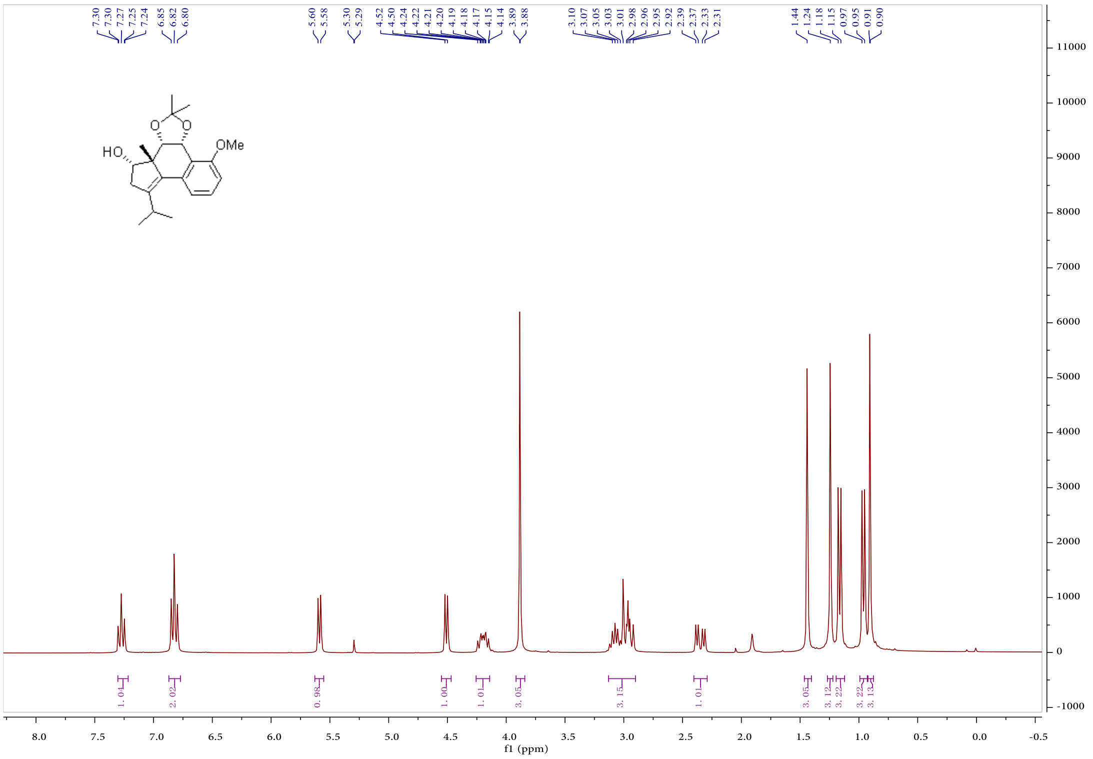

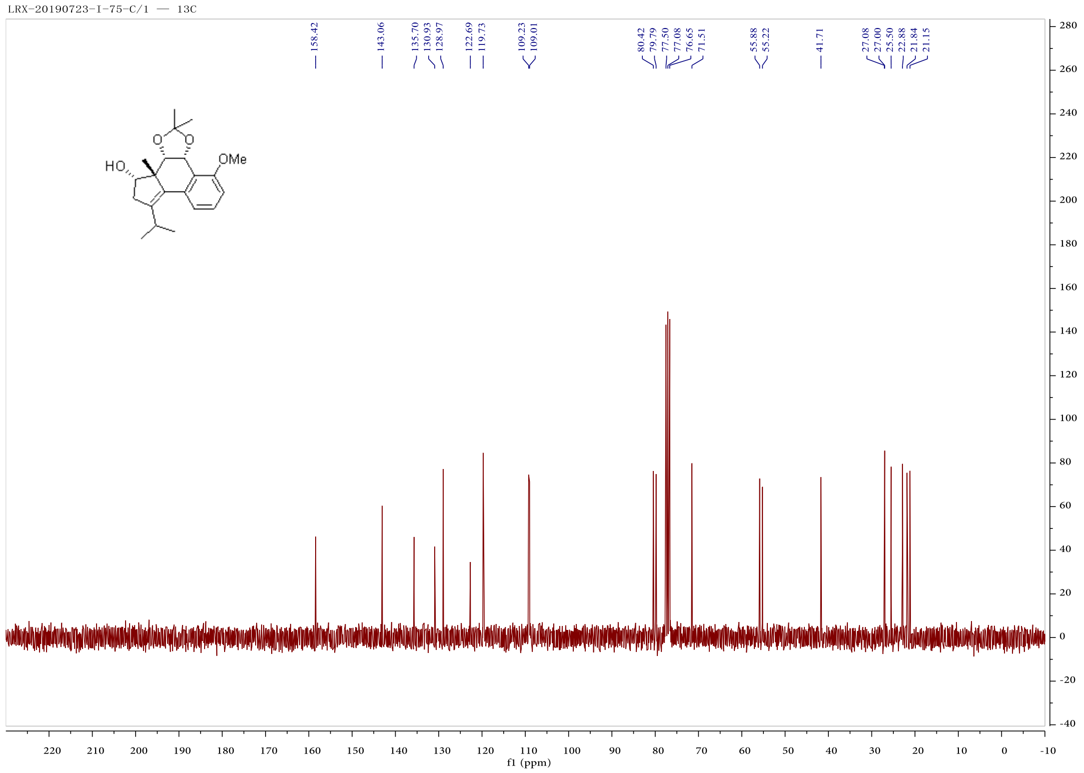
LRX-20190723-I-75-C/1 — 13C
158.42
143.06
135.70
130.93
128.97
122.69
119.73
109.23
109.01
80.42
79.79
77.50
77.08
76.65
71.51
55.88
55.22
41.71
27.08
27.00
25.50
22.88
21.84
21.15
HO
OMe
O
O
f1 (ppm)

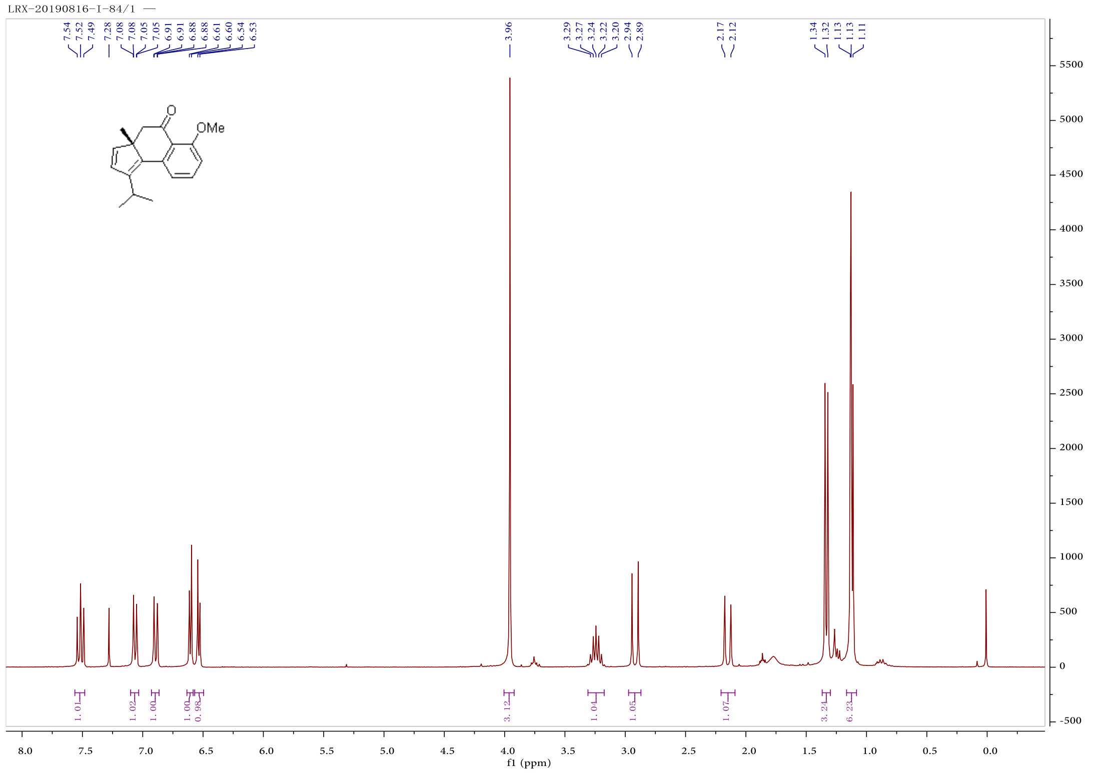
LRX-20190816-I-84/1 —
7.54
7.52
7.49
7.28
7.08
7.05
7.05
6.91
6.91
6.88
6.88
6.61
6.60
6.54
6.53
3.96
3.29
3.27
3.24
3.22
3.20
2.94
2.89
2.17
2.12
1.34
1.32
1.13
1.13
1.11
O
OMe
1.01
1.02
1.00
1.00
0.98
3.12
1.01
1.05
1.07
3.24
6.23
f1 (ppm)

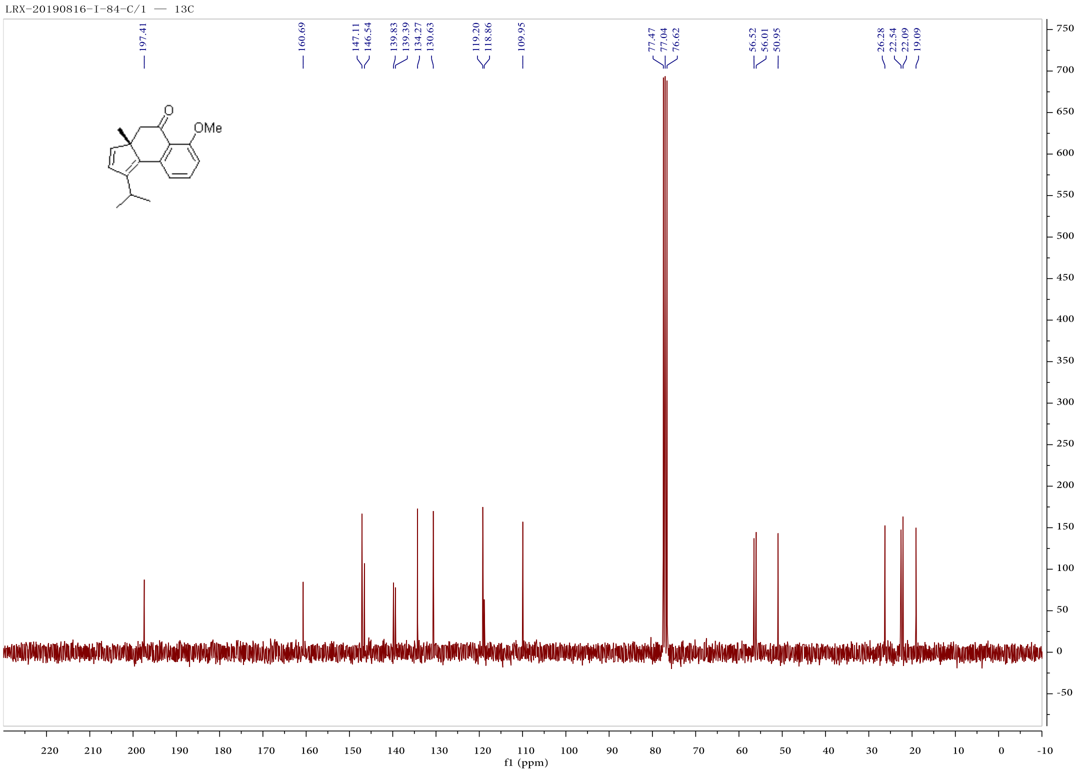
LRX-20190816-I-84-C/1 — 13C
197.41
160.69
147.11
146.54
139.83
139.39
134.27
130.63
119.20
118.86
109.95
77.47
77.04
76.62
56.52
56.01
50.95
26.28
22.54
22.09
19.09
OMe
f1 (ppm)

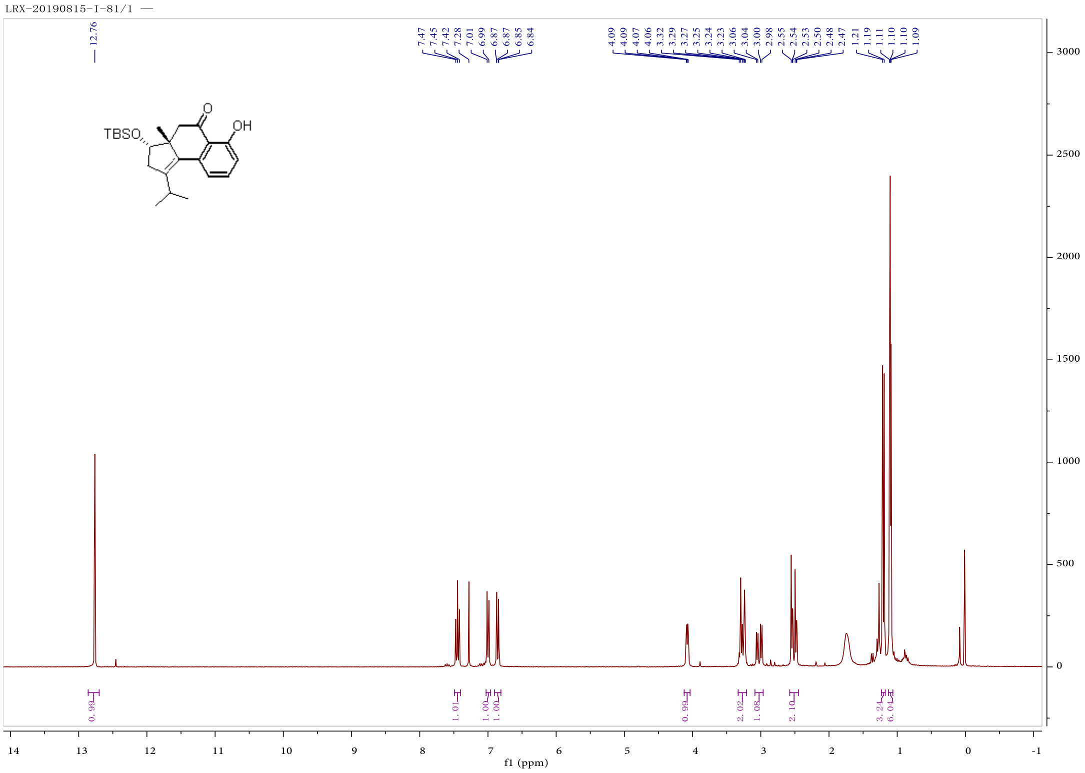
LRX-20190815-I-81/1 —
12.76
7.47
7.45
7.42
7.28
7.01
6.99
6.87
6.85
6.84
4.09
4.09
4.07
4.06
3.32
3.29
3.27
3.25
3.24
3.23
3.06
3.04
3.00
2.98
2.55
2.54
2.53
2.50
2.48
2.47
1.21
1.19
1.11
1.10
1.09
TBSO
OH
f1 (ppm)

LRX-20190815-I-81-C/1 — 13C

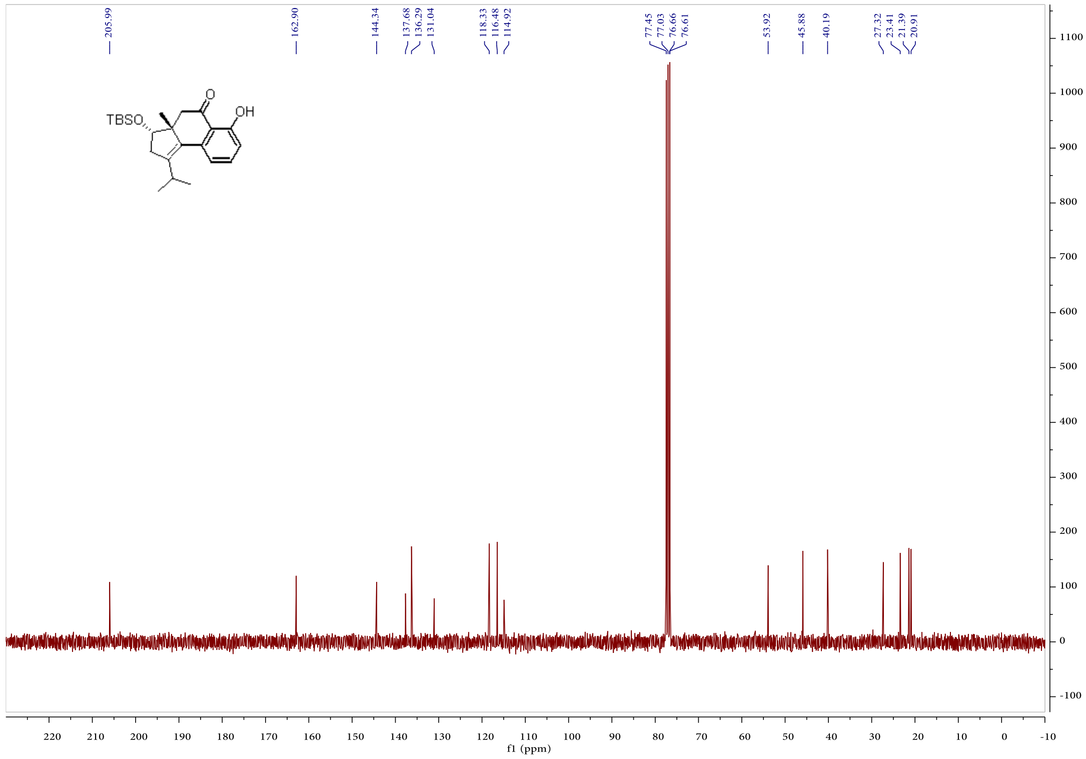

Supplement: Supplementary file 1 [file marinedrugs-18-00306-s001.pdf]
